# An asymmetric tetrabody is a potent and efficacious agonist of the erythropoietin receptor *in vitro* and *in vivo*

**DOI:** 10.1002/pro.70292

**Published:** 2025-09-17

**Authors:** Jarrett J. Adams, Levi L. Blazer, Jacky Chung, Minoo Karimi, Taylor Davidson, Heather A. Bruce, Alexander U. Singer, Ning Yang, Lia Cardarelli, Isabelle Pot, Luigi Colombo, Lily Jun‐shen Huang, Yue Ma, Stephen W. Michnick, Orson W. Moe, Sachdev S. Sidhu

**Affiliations:** ^1^ Anvil Institute for Systems Biologics Canada; ^2^ Simisco Biosciences USA; ^3^ EPOK Therapeutics Canada; ^4^ Université de Montréal Canada; ^5^ University of Texas Southwestern Medical Center USA

**Keywords:** agonist, anemia, antibody, diabody, dimer, drug design, EPO, EPOR, erythropoiesis, erythropoiesis‐stimulating agent, protein engineering, tetrabody

## Abstract

Erythropoietin (EPO) initiates EPO receptor (EPOR) signaling in hematopoietic cells by binding to an asymmetric EPOR dimer through two different sites. We engineered dimeric diabody‐Fc (Db‐Fc) fusion proteins that appeared to act as potent agonists of human EPOR in cell proliferation assays. However, detailed analysis of their oligomeric forms revealed that the predominant Db‐Fc species bound EPOR with high affinity but failed to induce cell proliferation. Instead, a minor oligomeric form, identified as a putative tetrabody (Tb) fused to two Fc domains (Tb‐Fc_2_), proved to be the minimal active form. The existence of a tetrameric agonist was further supported by crystallography, which revealed an asymmetric Tb structure. Additionally, the structure of an antigen‐binding fragment (Fab) bound to EPOR revealed an epitope distinct from the EPO binding sites, and structural modeling showed that engagement of two of the four binding sites on the Tb could form an asymmetric EPOR dimer nearly identical to the active conformation recruited by EPO. In a knock‐in mouse model, where mouse EPOR was replaced by human EPOR, purified Tb‐Fc_2_ stimulated erythropoiesis with greater potency, efficacy, and duration than darbepoetin, a recombinant EPO that is the leading therapeutic erythropoiesis‐stimulating agent (ESA). Collectively, these findings demonstrate that asymmetric tetravalent antibodies such as Tb‐Fc_2_ represent promising next‐generation ESAs that provide enhanced potency, efficacy, and durability. Moreover, they may reduce the oncogenic and cardiovascular risks associated with the pleiotropy of EPO.

## 1 INTRODUCTION

**FIGURE 1:**
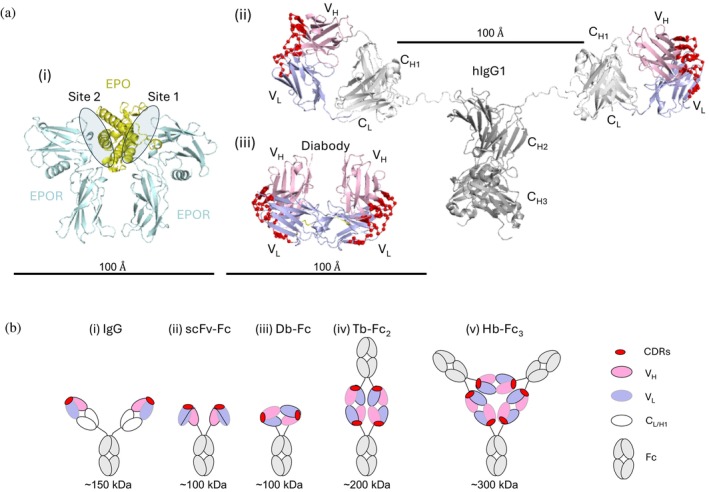
The EPO:(EPOR)2 complex and antibody formats. (a) Molecular renderings at the same scale of (i) the EPO:(EPOR)2 complex (PDB entry 1EER), (ii) a human IgG1 with extended reach (adapted from PDB entry 1HZH), and (iii) a human Db (adapted from PDB entry 9NWU). The following colors are used: EPO (yellow), EPOR (cyan), V_H_ (pink), V_L_ (lilac), C_L_ and C_H1_ (white), Fc (gray). Sites 1 and 2 on EPOR for binding to EPO are demarcated with shaded ovals. The Cα atoms of CDRs are shown as red spheres. (b) Various Ab formats. Cartoons with domains colored as in (a) are shown for the following: bivalent (i) IgG, (ii) scFv‐Fc, and (iii) Db‐Fc; (iv) tetravalent Tb‐Fc_2_; (v) hexavalent Hb‐Fc_3_. Illustrations are approximately 1/4 the scale shown in (a).

Erythropoietin (EPO) is a pleiotropic cytokine that mediates a wide range of cellular and physiological functions (Suresh et al., 2019). A principal and critical role of EPO is to regulate erythropoiesis, the generation of erythrocytes or red blood cells (RBCs) for oxygen transport throughout the body. Although several cytokines contribute to the maturation of RBCs, EPO is a master regulator triggering erythroid progenitors to develop into erythroblasts (Elliott et al., 2014). Activation of erythropoiesis is dependent on an asymmetric dimer of the erythropoietin receptor (EPOR) formed by engagement of a single EPO molecule on erythroid cells (Figure 1a). EPO first binds one EPOR through a high affinity site 1, and subsequently, recruits a second EPOR through a low affinity site 2. The active EPO:(EPOR)2 complex enables transphosphorylation and activation of the associated Janus (JAK) kinases, leading to activation of genes promoting cell survival and driving differentiation of the erythrocyte lineage (Broxmeyer, 2013).

In adults, EPO is mainly produced by interstitial cells of the kidney and circulates systemically as an endocrine hormone that stimulates cell production by acting on the early progenitors of the erythroid cell lineage (Suresh et al., 2019). Kidney diseases represent a state of dysfunction of this source of circulating EPO, which results in the anemia of chronic kidney disease (CKD) that is multi‐factorial in pathophysiology, with endocrine EPO deficiency playing a major role (Ramanath et al., 2012).

The purification of EPO, the cloning of its gene, and subsequent development of recombinant EPO (rEPO) revolutionized the treatment of anemia associated with EPO deficiency (Egrie, 1990; Lin et al., 1985; Sinclair, 2013). Although rEPO does not fully reverse all pathogenic components of CKD‐related anemia, it has markedly improved the ability to restore hematocrit levels in affected patients. However, the therapeutic success of EPO has been tempered by significant limitations. The optimal hematocrit target in CKD patients remains uncertain, and aiming for a “normal” hematocrit value has been associated with increased cerebrovascular risk (Coyne, 2012; Drüeke, 2018; Macdougall et al., 1990), perhaps because elevated hematocrit levels can cause hyperviscosity. EPO therapy may also activate non‐erythropoietic signaling pathways, contributing to adverse off‐target effects (Drüeke, 2018; Macdougall et al., 1990). It has been proposed that while erythropoiesis is stimulated by EPOR homodimer activation, at least some extra‐erythropoietic effects are mediated by EPO facilitating the formation of EPOR heterodimers with other receptors (Cantarelli et al., 2019; Ostrowski & Heinrich, 2018; Ramanath et al., 2012; Suresh et al., 2019). In particular, EPO has been proposed to bind to the β‐common cytokine receptor (β‐CR or CD131) (Hanazono et al., 1995; He et al., 2019; Jubinsky et al., 1997), although the existence of an EPOR:β‐CR heterodimer is still under debate (Cheung Tung Shing et al., 2018). EPO may also bind to the receptor tyrosine kinase EPHB4 through an as yet undefined mechanism (Li et al., 2015; Pradeep et al., 2015; Wang et al., 2022). These additional interactions may be especially prevalent with the high EPO levels that occur upon initial administration of recombinant EPO (Brines et al., 2008; Collino et al., 2015). The engagement of alternative receptors may be beneficial in some cases, such as the stimulation of cytoprotective pathways (Brines, 2010). However, EPO can also drive oncogenesis and the expansion of senescent tumor initiating populations, and at least in some cases, this may occur through the engagement of EPHB4 (Pradeep et al., 2015).

Recombinant human EPO and its variants have been used as erythropoiesis‐stimulating agents (ESAs) for the treatment of anemia for several decades (Eschbach et al., 1989). A hyperglycosylated EPO analog (darbepoetin) is currently the agent of choice in many clinical settings (Macdougall, 2024). Despite their clinical utility, EPO‐based ESAs present several limitations. First, EPO has a short half‐life, which mandates frequent administration. Even darbepoetin, with its extended half‐life, requires at least weekly dosing (Ibbotson & Goa, 2001). Second, EPO and its derivatives have pleiotropic biological actions, potentially mediated by heterodimeric receptors, as discussed above. These additional interactions may be particularly prominent during initial treatment phases when circulating rEPO levels are supra‐physiologic (Brines et al., 2008; Collino et al., 2015). These deleterious effects limit the patient populations that can benefit from EPO‐based ESAs, and therefore, an ESA that only activates the EPOR homodimer would be highly desirable as an alternative to pleiotropic EPO for the treatment of anemia.

Given the limitations associated with rEPO and its derivatives, there have been intense efforts to develop alternative ESAs that bind exclusively to EPOR with longer half‐lives *in vivo*. Notable examples of alternative ESAs include designed ankyrin repeats and dimeric synthetic peptides. Although these agents have shown some promising efficacy *in vitro*, they are non‐natural polypeptides that are foreign to the human body and carry risks of unexpected complications *in vivo*. The potential severity of these risks is exemplified by peginesatide, a pegylated dimeric peptide agonist of EPOR that gained clinical approval for the treatment of anemia. Despite its initial promise, peginesatide was withdrawn from the market following reports of fatal hypersensitivity reactions (Gupta & Wish, 2018; Kaushik & Yaqoob, 2013). This case highlights the need for novel ESAs that not only exhibit high potency and efficacy but also maintain a favorable safety profile by minimizing immunogenicity and off‐target effects.

Antibodies (Abs) are a dominant class of therapeutic agents with broad applications across many disease indications (Crescioli et al., 2025). Most importantly, therapeutic Abs resemble natural Abs that are present at high concentrations in human serum, and thus, they are well tolerated in patients and exhibit long half‐lives and low immunogenicity.

Given these favorable properties, intensive efforts have been made by many groups to engineer bivalent IgGs as agonists of EPOR. However, the two antigen‐binding fragment (Fab) arms of an IgG are much larger and more flexible than EPO (Figure 1b(i)), and thus, they cannot bring two EPOR molecules into the close and precise configuration necessary for effective signal transduction (Liu et al., 2007). To address this limitation, single‐chain variable fragments (scFvs) fused to an Fc have been engineered to reduce the size of the binding arms (Figure 1b(ii)), yet they still fail to accurately mimic the geometry of EPO and show limited agonist activity (Zhang et al., 2012). Most recently, dimeric diabodies (Dbs), in which the two binding sites are closely linked in a more rigid structure, have been explored as agonists of EPOR, but structural studies have shown that even the binding sites of Dbs are too far apart (Figure 1b(iii)) to enable dimerization of EPOR in a conformation in which the juxtamembrane regions are in the close proximity induced by EPO binding (Moraga et al., 2015). Complicating these efforts further, IgGs, scFvs, and Dbs tend to oligomerize into higher‐order species, raising uncertainty as to whether the observed agonist activities arise from the intended bivalent form or from unintended oligomeric forms. Despite many attempts and the promising *in vitro* efficacy of various Ab agonists of EPOR, it remains unclear whether a dimeric Ab in any format can activate EPOR with sufficient potency and efficacy to induce efficient erythropoiesis *in vivo*. Consequently, no EPOR agonist Ab has entered clinical trials or gained regulatory approval.

Despite the discouraging history described above, we revisited the question of whether Abs can be engineered to be EPOR agonists that are sufficiently effective to function as therapeutic agents to stimulate erythropoiesis *in vivo*. We constructed a phage‐displayed Db library using a highly stable human framework that has been used for the development of many successful Ab biologics. Following selections for binders to human EPOR, we screened many Dbs fused to an Fc (Db‐Fc, Figure 1b(iii)) and identified potential agonists that induced proliferation of cells whose growth depends on stimulation of EPOR. However, close inspection of the oligomeric states of these agonists revealed that the major dimeric Db‐Fc form bound tightly to EPOR but lacked agonist activity. Instead, a minor form representing a putative tetrabody (Tb) fused to two Fc domains (Tb‐Fc_2_, Figure 1b(iv)) emerged as the minimal active form capable of inducing EPOR signaling both *in vitro* and *in vivo*.

This supposition was supported by crystallography studies that elucidated the structure of an asymmetric Tb agonist that binds to an epitope on EPOR distinct from that of EPO. Structural modeling showed that the asymmetric nature of the Tb and its novel epitope on EPOR enables two of the four binding sites to recruit an asymmetric EPOR dimer nearly identical to the active conformation recruited by EPO. Collectively, these findings support the concept that asymmetric, tetravalent Abs represent a promising class of next‐generation ESAs capable of selectively activating EPOR with high potency while avoiding engagement of other receptors that have been implicated in tumorigenesis and other adverse effects.

## 2 RESULTS

### 2.1 Construction of a phage‐displayed Db library

Minimal antigen‐binding fragments can be engineered by fusing the C‐terminus of the light‐chain variable (V_L_) domain to the N‐terminus of the heavy‐chain variable (V_H_) domain to form single‐chain variable fragments (scFvs). The oligomeric state of these V_L_–V_H_ fusions depends on the length of the intra‐domain linker, with shorter linkers favoring larger oligomers. A detailed study with a model V_L_–V_H_ fusion showed that linkers containing >12, 12–3, or 2–0 residues favored the formation of monomeric (scFv), dimeric (Db), or trimeric (triabody) and tetrameric (Tb) quaternary structures, respectively (Todorovska et al., 2001).

**FIGURE 2:**
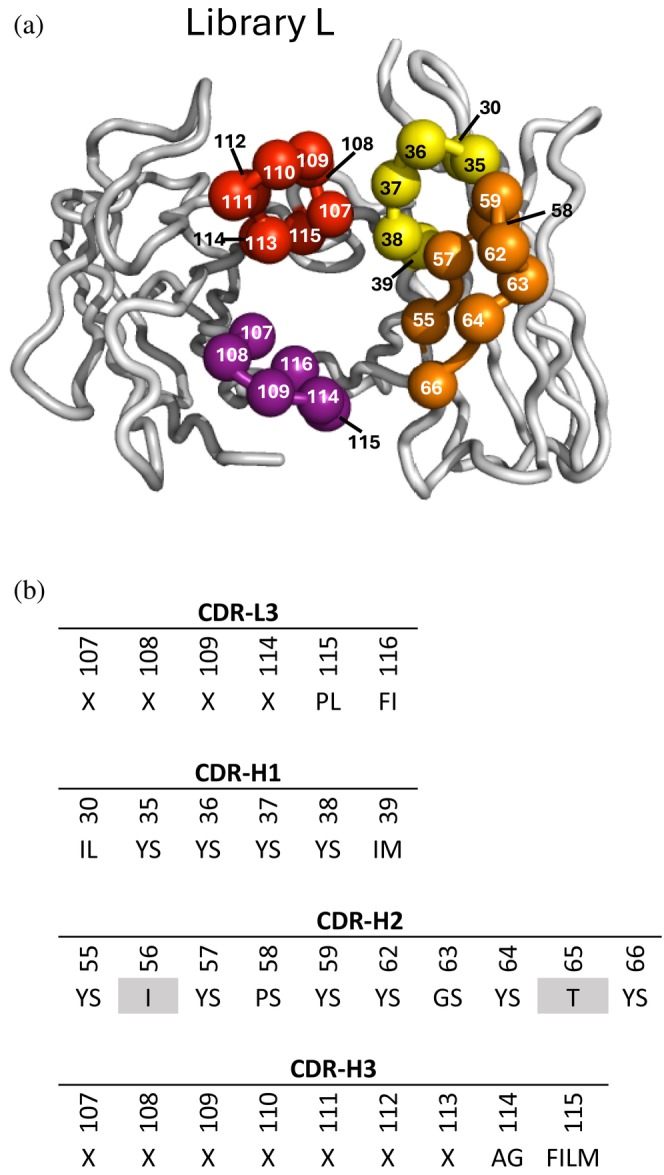
Phage‐displayed Db library design. (a) The backbones of the V_L_ and V_H_ domains are shown as tubes. The frameworks are colored gray, and the CDR loops are colored as follows: L3 (purple), H1 (yellow), H2 (orange), H3 (red). Spheres represent positions that were diversified. The figure was adapted from the structure coordinates of Fab 4D5 (PDB entry 1FVC). (b) CDR diversity design. Positions shaded in gray were fixed, and at each diversified position, the allowed amino acids are denoted by the single‐letter code. X denotes a mixture of nine amino acids (Y, S, G, A, F, W, H, P, V) introduced at proportions described previously (Persson et al., 2013). The lengths of CDRs L3 and H3 were varied by replacing the positions denoted by X with 3–7 or 1–17 degenerate codons, respectively. Positions are numbered according to the IMGT scheme (Persson et al., 2013).

To construct a phage‐displayed Db library, we modified a phagemid vector encoding a human scFv by introducing a Gly_5_ linker between the V_L_ and V_H_ domains. This configuration produced an open reading frame encoding a putative Db linked to the C‐terminal domain of the M13 bacteriophage gene‐3 minor coat protein with a modified IgG hinge sequence (Figure S1). The phagemid was used to construct a Db library in which four of the six complementarity‐determining regions (CDRs) were diversified with a tailored strategy. Specifically, the three heavy chain CDRs and CDR‐L3 were diversified as described for a Fab library (Persson et al., 2013), although with reduced length diversity for CDR‐H3 (Figure 2). The resulting library contained 4.2 × 10^9^ unique clones, and DNA sequencing revealed that 50% of these were diversified in all 4 CDRs.

### 2.2 Screening for EPOR agonists

Phage pools representing the Db library were cycled through rounds of binding selections on the target protein consisting of the human EPOR ectodomain (ECD) fused to a human Fc domain (EPOR‐Fc). Following five rounds of selections, individual clones were assessed for specific binding by phage enzyme‐linked immunosorbent assays (ELISAs) with immobilized EPOR‐Fc or bovine serum albumin (BSA) as a negative control. DNA sequencing of clones that bound to EPOR‐Fc but not BSA revealed 26 unique clones.

The 26 clones were purified following recombinant expression in mammalian cells in a Db‐Fc fusion protein format. Each Db‐Fc was assessed by flow cytometry for binding to TF‐1 cells, which are derived from a human erythroleukemia, display endogenous EPOR, and only grow in the presence of either EPO, granulocyte‐macrophage colony‐stimulating factor (GMCSF), or interleukin 3 (IL‐3) (Kitamura et al., 1989). Fourteen of the 26 Db‐Fc proteins exhibited binding to the TF‐1 cells (Figure S2) and these were subjected to further characterization.

**FIGURE 3:**
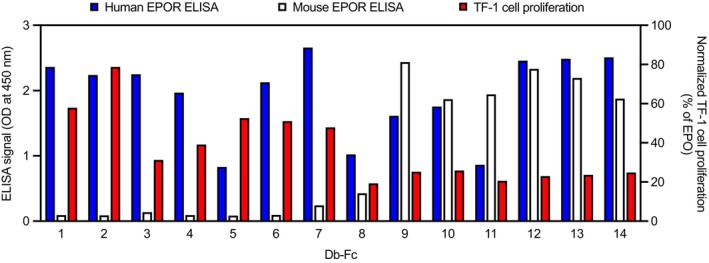
Binding and agonist activity of Db‐Fc proteins. Db‐Fc proteins (*x*‐axis) were assessed by ELISA (*y*‐axis, left) for binding to immobilized human (blue) or mouse (white) EPOR‐Fc and for their effects on TF‐1 cell proliferation (*y*‐axis, right) relative to EPO (red).

The 14 Db‐Fc proteins were tested for binding to human or mouse EPOR‐Fc by ELISA, and as expected, they all bound to human EPOR‐Fc but only 7 also bound strongly to mouse EPOR (mEPOR) (Figure 3). Next, we addressed the critical question of whether the Db‐Fc proteins could act as agonists of EPOR signaling and induce proliferation of TF‐1 cells. Only seven (Db‐Fc 1–7) induced significant increases in TF‐1 cell proliferation (Figure 3), and notably, none of these bound to mEPOR, whereas the 7 Db‐Fc that did not induce proliferation bound to mEPOR (D‐Fc 8–14). Db‐Fc 1 and 2 demonstrated the greatest proliferative activity and were selected for further biochemical and functional characterization.

### 2.3 Characterization of naïve agonists of EPOR

**FIGURE 4:**
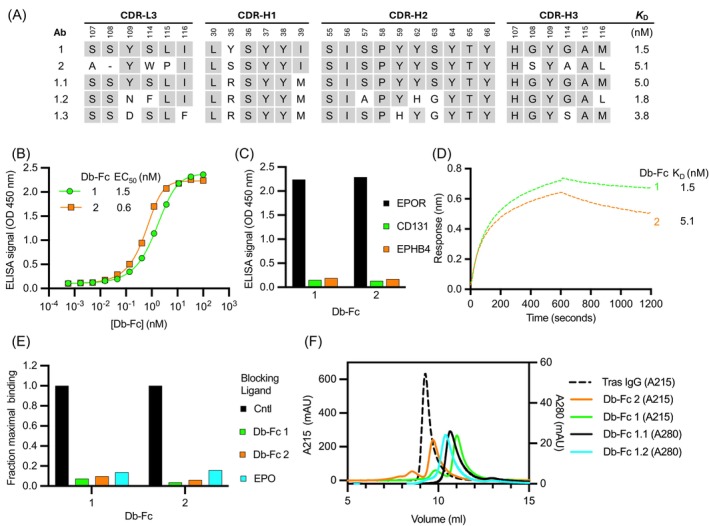
Characterization of EPOR agonists. (a) Sequences of anti‐EPOR Abs are shown for the CDR positions that were diversified in the naïve phage‐displayed library and are numbered according to the IMGT nomenclature. Identities with sequence 1 are shaded gray. The dissociation constants (*K*_D_) determined by BLI are shown at the right. (b) Binding (*y*‐axis) of immobilized EPOR‐Fc to serial dilutions of Db‐Fc protein (*x*‐axis) 1 (green circles) or 2 (orange squares) assessed by ELISA. (c) ELISAs for binding (*y*‐axis) of Db‐Fc protein (*x*‐axis) to His‐tagged ECDs of EPOR (black), CD131 (green), or EPHB4 (orange). (d) BLI assays for detection of 100 nM solution‐phase Db‐Fc protein binding to immobilized EPOR‐Fc. Binding response (*y*‐axis) was recorded for 600 s of association followed by 600 s of dissociation (*x*‐axis) for Db‐Fc 1 (green) and 2 (orange). (e) Binding of Db‐Fc (*x*‐axis) to EPOR in the absence of competing ligand (black) or in the presence of Db‐Fc 1 (green), Db‐Fc 2 (orange), or EPO (cyan). The binding signal (*y*‐axis) was normalized to the binding signal in the absence of competing ligand. Competition with Db‐Fc or EPO was determined by BLI or ELISA, respectively. (f) SEC of Db‐Fc proteins. Proteins were purified by affinity chromatography with Protein‐A resin and applied to a Tosoh TSK gel filtration column at 1 mg/mL in PBS. The column was run with PBS (*x*‐axis) and absorption was monitored at either 215 or 280 nm wavelength (*y*‐axis, left or right, respectively). Trastuzumab IgG (dashed black) was run as a control for comparison with Db‐Fc 2 (orange), Db‐Fc 1 (green), Db‐Fc 1.1 (black), and Db‐Fc 1.2 (cyan).

Despite being isolated from a naïve library and thus not being related to each other by design, Dbs 1 and 2 showed high homology in their CDR sequences (Figure 4a), suggesting that they bind to EPOR in a similar manner. ELISAs showed that Db‐Fcs 1 and 2 bound to immobilized EPOR‐Fc with high apparent affinities (EC_50_ = 1.5 or 0.6 nM, respectively, Figure 4b). Notably, neither Db‐Fc bound detectably to EPHB4 or CD131 (Figure 4c), two other receptors that have been reported to interact with EPO to mediate alternative signaling pathways (Debeljak et al., 2014). Kinetic analysis using biolayer interferometry (BLI) confirmed the nanomolar affinity of solution‐phase Db‐Fc 1 and 2 for immobilized EPOR‐Fc (*K*_D_ = 1.5 or 5.1 nM, respectively, Figure 4d and S3). A BLI assay also showed that Db‐Fcs 1 and 2 competed for binding to immobilized EPOR‐Fc, and a competition ELISA showed that the binding of both Db‐Fcs to immobilized EPOR‐Fc was blocked by EPO (Figure 4e). Taken together, these results showed that Db‐Fcs 1 and 2 bind with high affinities to EPOR but not to other receptors that may bind to EPO, and moreover, they compete with each other and with EPO for binding to EPOR, suggesting that they bind to similar epitopes that overlap with the high‐affinity EPO‐binding site on EPOR.

**FIGURE 5:**
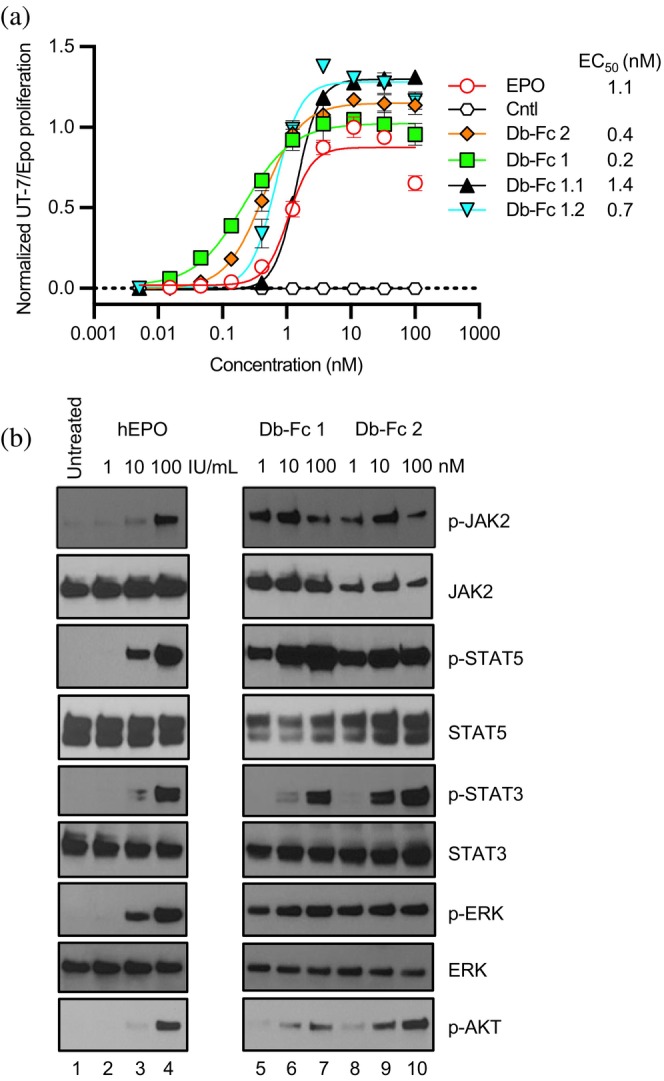
UT‐7/Epo cell proliferation assays for EPOR agonist activity. (a) UT‐7/Epo cell proliferation (y‐axis) was monitored in the presence of various concentrations (*x*‐axis) of positive control EPO (unfilled red circles), a negative isotype control Ab (unfilled black hexagons), or the following Db‐Fc proteins: 2 (filled orange diamonds), 1 (filled green squares), 1.1 (filled black triangles), 1.2 (filled inverted cyan triangles). Cell numbers and viability were assessed by ATP/luciferase luminescence. (B) Immunoblot analysis of intracellular effectors of EPOR signaling. As indicated above each lane, UT‐7/Epo cells were either untreated (lane 1) or treated with various concentrations of EPO (lanes 2–4), Db‐Fc 1 (lanes 5–7), or Db‐Fc 2 (lanes 8–10). Membranes were blotted for the unphosphorylated and phosphorylated (p) forms of the following proteins: JAK‐2, STAT5, STAT3, ERK, AKT.

Next, we used cell‐based assays to assess the functional effects of Db‐Fcs 1 and 2 on the signaling activity of EPOR. We used UT‐7/Epo cells, which were derived from the human leukemia line UT‐7 by long‐term culture in the presence of EPO. Unlike UT‐7 and TF‐1 cells, UT‐7/Epo cells have lost the ability to grow in the presence of GMCSF or IL‐3 and are absolutely dependent on EPO (Komatsu et al., 1993). Consequently, UT‐7/Epo cells are considered to be more committed to the erythroid lineage than either UT‐7 or TF‐1 cells and are ideal for assessing proliferation induced by EPO mimetics through EPOR. Db‐Fcs 1 and 2 were as efficacious and more potent than EPO in stimulating the proliferation of UT‐7/Epo cells (EC_50_ = 0.2, 0.4, or 1.1 nM, respectively, Figure 5a). We also used immunoblotting to assess the phosphorylation of EPOR effector pathways (Figure 5b). As observed by others (Moraga et al., 2015; Shi et al., 2010), EPO induced the phosphorylation of the canonical targets JAK2, STAT3, STAT5, ERK, and AKT in a dose‐dependent manner. Importantly, Db‐Fcs 1 and 2 both induced the phosphorylation of all 5 signaling molecules in a manner very similar to EPO, and Db‐Fc 1 was particularly effective. Taken together, these results showed that our two lead Db‐Fcs are agonists of EPOR that induce cell proliferation and phosphorylation events very similar to those induced by EPO.

### 2.4 Development of optimized EPOR agonists

Our goal was to determine whether dimeric Dbs could act as effective agonists of EPOR. Thus, we designed our V_L_–V_H_ fusions with a five‐residue linker that favors Dbs over scFvs and higher‐order oligomers, and we added a dimeric Fc to further reinforce a dimeric structure. However, even minor levels of higher‐order oligomers that act as potent agonists could complicate the assessment of agonist activity. Consequently, we used size‐exclusion chromatography (SEC) to analyze the oligomeric states of Db‐Fcs 1 and 2 (Figure 4f). This analysis revealed that both proteins eluted predominantly as single peaks, presumably representing the dimeric Db‐Fc species (Figure 1b(iii)), but each also displayed a minor, earlier‐eluting peak, consistent with a higher molecular weight species. We hypothesize that these species represent tetrameric Dbs fused to two Fc domains (Tb‐Fc_2_, Figure 1b(iv)), which may contribute disproportionately to EPOR activation.

To explore whether sequence changes in V_L_–V_H_ 1 could alter the proportion of protein that eluted as a dimeric Db‐Fc, we constructed a phage‐displayed sub‐library based on the sequence of V_L_–V_H_ 1, in which the 3 heavy‐chain CDRs were subjected to a “soft randomization” process such that approximately half of the codons in the variant library were wild‐type and the other half were random substitutions. The library was subjected to binding selections for EPOR, specific binding clones were sequenced, and Fc fusions were screened by SEC to identify those with the cleanest elution profiles. Through this process, we identified variant 1.1 (Figure 4a), which contained two substitutions in CDR‐H1 relative to the parental sequence (Y^35H^R/I^39H^M) and eluted almost completely (>99%) as a monodisperse peak corresponding to the Db‐Fc form (Figure 4f). However, assessment of binding to immobilized EPOR by BLI (Figure 4a and S3) revealed that the affinity of Db‐Fc 1.1 was compromised compared with Db‐Fc 1 (*K*_D_ = 5.0 or 1.5 nM, respectively), consistent with a modest decrease in potency in UT‐7/Epo cell proliferation assays (Figure 5a).

To enhance affinity while retaining biophysical homogeneity, we next constructed a phage‐displayed library based on the sequence of variant 1.1 in which CDRs H2, H3, and L3 were subjected to soft randomization. Following selection for binding to EPOR, a panel of variants was purified in the Db‐Fc format and screened for mono‐dispersity by SEC and affinity by BLI. This effort yielded variant 1.2 (Figure 4a), which contained a total of six substitutions relative to variant 1.1, exhibited a nearly homogenous Db‐Fc peak (Figure 4f), bound to EPOR with high affinity (*K*_D_ = 1.8 nM, Figure 4a and S3), and was highly potent in UT‐7/Epo cell proliferation assays (Figure 5a).

### 2.5 Effects of V_L_ –V_H_ oligomerization on EPOR agonist activity

**FIGURE 6:**
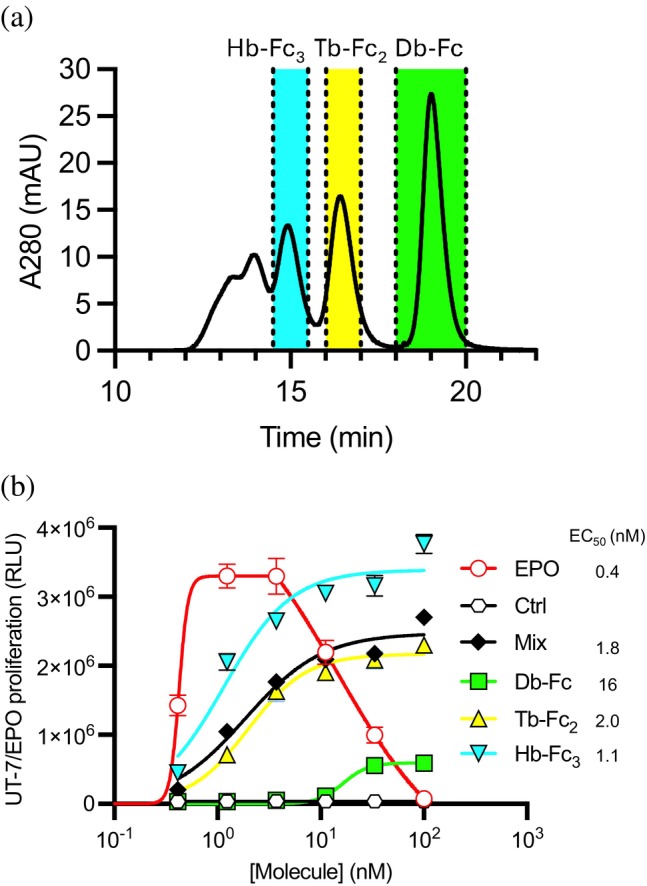
Effects of V_L_–V_H_ 1.3 oligomerization on EPOR agonist activity. (A) SEC of Db‐Fc 1.3 on a preparative Superdex 200 column. Protein peaks were collected as 3 fractions indicated by dotted lines representing putative Db‐Fc (green), Tb‐Fc_2_ (yellow), or Hb‐Fc_3_ (cyan). (B) UT‐7/Epo cell proliferation assay. Proliferation (*y*‐axis) was monitored in the presence of various concentrations (*x*‐axis) of positive control EPO (unfilled red circles), a negative isotype control Ab (unfilled black hexagons), the unfractionated mixture of Db‐Fc (filled black diamonds), or the following purified fractions: Db‐Fc (filled green squares), Tb‐Fc_2_ (filled yellow triangles), Hb‐Fc_3_ (filled inverted cyan triangles). Cell numbers and viability were assessed by ATP/luciferase luminescence.

The progression towards optimized agonists of EPOR yielded several variants with improved biophysical profiles, especially enhanced mono‐dispersity in SEC assays. For example, variants 1.1 and 1.2 exhibited SEC profiles that showed that a greater percentage of the protein was present in the Db‐Fc form compared with the parental variant 1. However, other variants exhibited greater proportions of oligomers compared with variant 1. In particular, variant 1.3 contained seven substitutions relative to variant 1 (Figure 4a), but in this case, the protein exhibited a “ladder‐like” elution profile with the Db‐Fc peak being dominant, but two faster‐eluting peaks, likely representing larger oligomers, also being prominent and distinct (Figure 6a).

The presence of these distinct higher‐order peaks in variant 1.3 enabled dissection of their individual contributions to EPOR agonism. By preparative SEC, we were able to purify fractions representing putative dimeric Db‐Fc (peak 1, Figure 1b(iii)), putative tetrameric Tb‐Fc_2_ (peak 2, Figure 1b(iv)), and a putative hexabody fused to three Fc domains (Hb‐Fc_3_, peak 3, Figure 1b(v)).

We assessed the activity of each fraction in the UT‐7/Epo cell proliferation assay (Figure 6b). Prior to fractionation by preparative SEC, the Db‐Fc_mix_ showed good potency (EC_50_ = 1.8 nM) and efficacy (maximum response = 2.5 × 10^6^ RLU), compared with EPO (EC_50_ = 0.4 nM and maximum response = 3.2 × 10^6^ RLU). In contrast, the purified Db‐Fc fraction (Figure 1b(iii)) showed low potency (EC_50_ = 16 nM) and efficacy (maximum response = 6 × 10^5^ RLU), and even this low activity may have been due to higher‐order oligomer impurities. Notably, the Tb‐Fc_2_ peak (Figure 1b(iv)) showed potency (EC_50_ = 2.0 nM) and efficacy (maximum response = 2.2 × 10^6^ RLU) that were nearly identical to those of the mixture. Finally, the Hb‐Fc_3_ peak (Figure 1b(v)) showed potency (EC_50_ = 1.1 nM) and efficacy (maximum response = 3.4 × 10^6^ RLU) that exceeded those of the mixture and were comparable to those of EPO.

As observed previously (Kim et al., 2017; Zhang et al., 2009), a hook effect was observed in the cell proliferation response to EPO, although the concentration of the hook varied across assays, limiting our ability to benchmark activity to EPO from UT‐7/EPO cell proliferation assays alone. This phenomenon is due to the fact that EPO contains a high‐affinity site 1 that binds to EPOR first and a low‐affinity site 2 that engages a second EPOR to form the active signaling complex. At high concentrations of EPO, site 1 engagement dominates and obfuscates site 2 binding so that each EPO associates with only one EPOR, generating an inactive complex (Lacy et al., 2008). In contrast, we saw no evidence of a hook effect in any of the Ab preparations, and this is consistent with all binding sites in these molecules having equivalent affinities so that avidity causes mainly multivalent binding. Collectively, these data indicate that while bivalent Db‐Fc proteins are not EPOR agonists, higher‐order oligomeric forms such as Tb‐Fc_2_ and Hb‐Fc_3_ effectively cluster EPOR and drive potent proliferative signaling. These findings underscore the importance of Ab valency and spatial architecture in fine‐tuning receptor activation and suggest that engineered multivalent Abs may serve as next‐generation ESAs with optimized efficacy and selectivity.

### 2.6 Assessment of EPOR agonist activity *in vivo*

**FIGURE 7:**
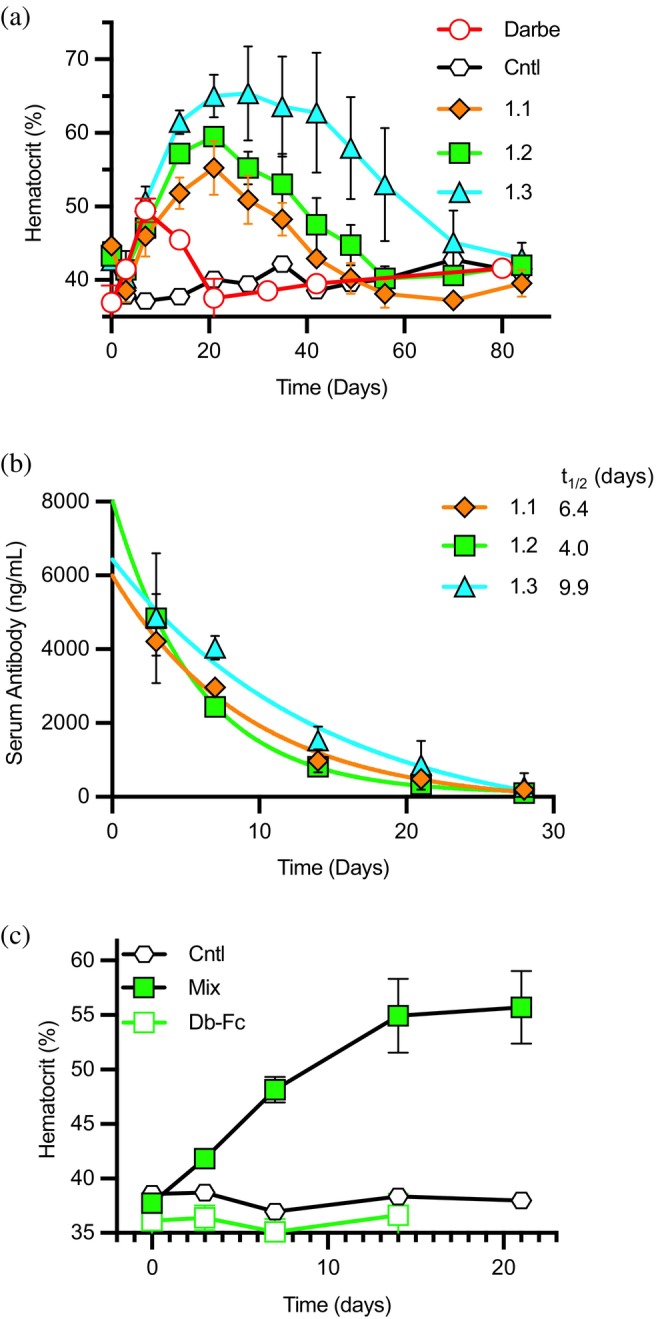
Assessment of EPOR agonist activity *in vivo*. (a) *In vivo* effects on hematocrit in humanized EPOR mice treated by intraperitoneal injection with positive control darbepoetin (1 mg/kg, unfilled red circles), a negative isotype control Ab (2 mg/kg, unfilled black hexagons), or unfractionated Db‐Fc_mix_ 1.1 (2 mg/kg, filled orange diamonds), 1.2 (2 mg/kg, filled green squares), or 1.3 (2 mg/kg, filled cyan triangles). Peripheral blood hematocrit (*y*‐axis) was assayed at various time points (*x*‐axis). (b) Serum concentrations (*y*‐axis) of Abs shown in (a) at various time points following injection (*x*‐axis). The half‐life (*t*_1/2_) was calculated for each curve fit. (c) *In vivo* effects on hematocrit in humanized EPOR mice treated by intraperitoneal injection with a negative control Ab (2 mg/kg, unfilled black octagons), unfractionated mixture of Db‐Fc 1.2 (2 mg/kg, filled green squares), or purified Db‐Fc 1.2 (2 mg/kg, unfilled green squares).

To evaluate the erythropoietic activity of our EPOR agonists in a physiological context, we tested the activities of the Db‐Fc variants *in vivo*, using knock‐in mice engineered to express the human EPOR gene (Divoky & Prchal, 2002; Lacy et al., 2008; Liu et al., 2007) (Figure 7a). The Abs were purified by protein‐A affinity chromatography but were not further fractionated by preparative SEC to isolate individual oligomeric forms prior to injection. Consistent with higher order oligomers being the active species, injection with the most heterogenous variant 1.3 resulted in the greatest increase in hematocrit compared with the more monodisperse variants 1.1 and 1.2. Notably, the maximum response and durability of response for all the Ab variants significantly exceeded that for the positive control darbepoetin, and remarkably, mice injected with variant 1.3 exhibited significantly sustained, elevated hematocrit levels more than 2 months post‐injection. In all cases, hematocrit levels eventually returned to baseline, demonstrating reversibility of the response. Pharmacokinetic analysis revealed that serum levels of all three Ab variants remained detectable more than 1 week post‐injection, with estimated half‐lives ranging from 4 to 10 days (Figure 7b).

For variant 1.2, we compared the *in vivo* activity of the unfractionated mixture with that of the major Db‐Fc fraction purified by preparative SEC (Figure S4). Injection of the mixture again caused a rapid and strong increase in hematocrit levels. In contrast, injection of the Db‐Fc fraction or a negative control Ab did not alter hematocrit levels (Figure 7c). Consistent with the cell proliferation results, the *in vivo* studies confirmed that the bivalent Db‐Fc does not activate EPOR, but rather, higher‐order oligomers are responsible for the potent and durable erythropoietic effects observed in human EPOR knock‐in mice.

### 2.7 Crystal structure of an asymmetric tetrabody

**FIGURE 8:**
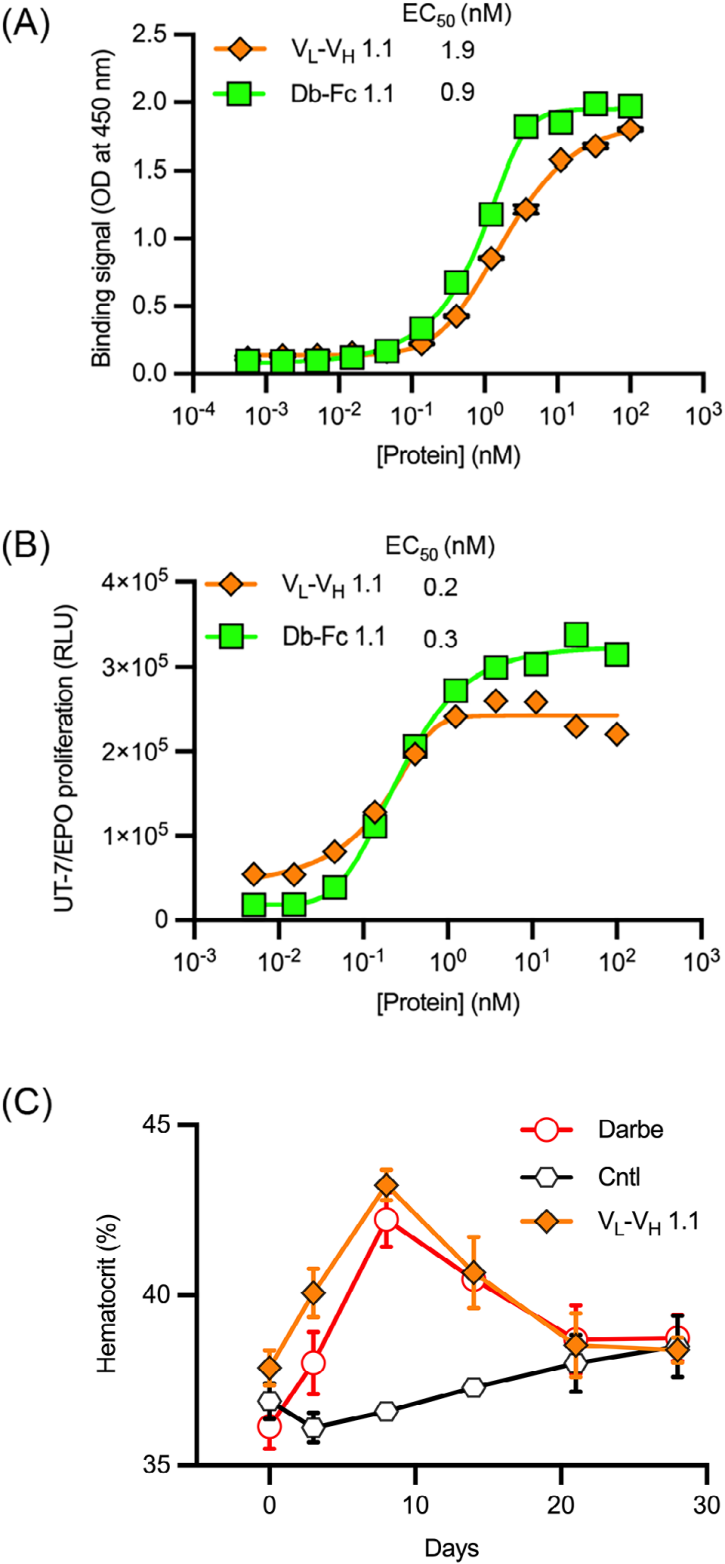
Binding and activity of V_L_–V_H_ 1.1 without or with an Fc domain. (a) Binding (*y*‐axis) of immobilized EPOR‐Fc to serial dilutions of V_L_–V_H_ 1.1 (*x*‐axis) without (orange diamonds) or with (green squares) an Fc domain. (b) UT‐7/Epo cell proliferation (*y*‐axis) induced by serial dilutions of V_L_–V_H_ 1.1 (*x*‐axis) without (orange diamonds) or with (green squares) an Fc domain. (c) *In vivo* effects on hematocrit in humanized EPOR mice treated by intraperitoneal injection with positive control darbepoetin (1 mg/kg, unfilled red circles), a negative isotype control Ab (2 mg/kg, unfilled black hexagons), or V_L_–V_H_ 1.1 without an Fc domain (2 mg/kg, filled orange diamonds). Peripheral blood hematocrit (*y*‐axis) was assayed at various time points (*x*‐axis).

To better understand the basis for V_L_–V_H_ oligomerization and activity, we produced V_L_–V_H_ 1.1 without an Fc domain. The V_L_–V_H_ protein bound tightly to immobilized EPOR‐Fc by ELISA (Figure 8a), and it was highly active in the UT‐7/Epo cell proliferation assay (Figure 8b). In both assays, the activity profile of the V_L_–V_H_ mirrored that of the Db‐Fc 1.1, showing that the Fc domain is not required for agonist activity. Moreover, in the *in vivo* mouse model, administration of the V_L_–V_H_ protein caused an increase in hematocrit levels similar to that induced by darbepoetin (Figure 8c). Taken together, these results showed that, even without an Fc domain, spontaneous oligomers of V_L_–V_H_ act as autonomous agonists of EPOR *in vitro* and *in vivo*, but the absence of the Fc domain does reduce the half‐life and thus the duration of response *in vivo*.

**FIGURE 9:**
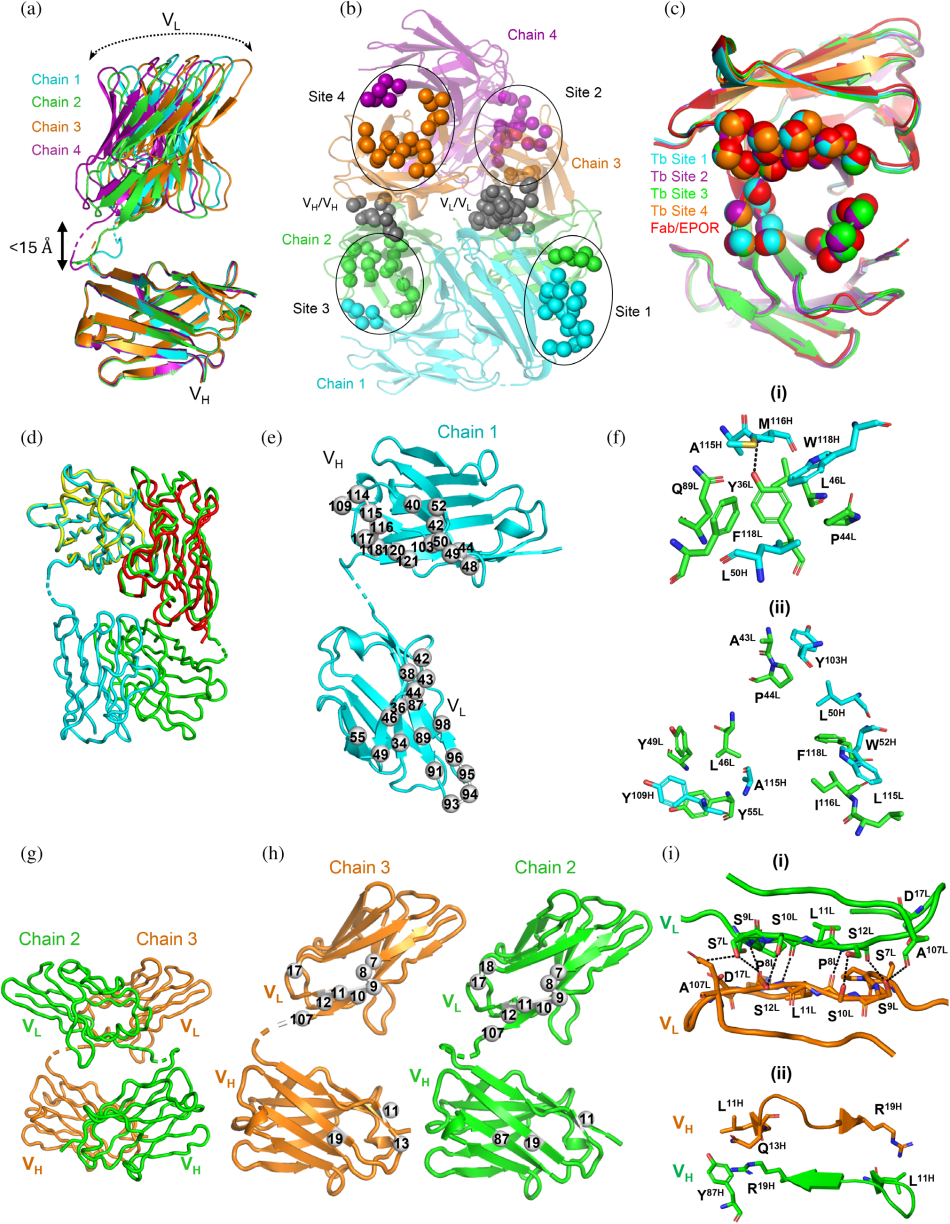
The asymmetric crystallographic Tb formed by V_L_–V_H_ 1.1. (A) Superpositions of the four V_L_–V_H_ chains aligned on V_H_ chains. Chains 1, 2, 3, and 4 are colored cyan, green, orange, or purple, respectively. (b) Crystal structure of the asymmetric unit of V_L_–V_H_ 1.1. CDR positions that were diversified in the library design are shown as spheres colored according to the chain color, and the four antigen‐binding sites are labeled and demarcated with ovals. The residues that make contacts (<4 Å) at the V_L_/V_L_ and V_H_/V_H_ interfaces and stabilize the Tb are shown as gray Cα spheres. (c) Superpositions of the four Tb antigen‐binding sites (cyan, purple, green, orange) with the Fab 1.1 antigen‐binding site when bound to EPOR (red, see Figure 10). Residues in the antigen‐binding sites are shown as Cα spheres. Root mean square deviations of the Fab antigen‐binding site are <0.5 Å from the Tb antigen‐binding sites. (d) The Db composed of V_L_–V_H_ 1.1 chains 1 (cyan) and 2 (green) superposed with the 4D5 Fv (pdb entry 1FVC) colored red (V_L_) and yellow (V_H_). (e) Chain 1 with residues that make contact with chain 2 in the V_L_/V_H_ interfaces of the Db depicted as gray Cα spheres numbered according to the IMGT database (Lefranc et al., 2003). (f) Molecular details of the contacts between the V_H_ domain of chain 1 and the V_L_ domain of chain 2. (i) The hydrogen bond (dashed line) between the sidechain of Tyr^36L^ and the mainchain of Met^116H^ and surrounding hydrophobic interactions. (ii) Additional hydrophobic interactions between residues in the framework and CDRs H3 and L3. (g) The unconventional dimer formed by chains 2 and 3. (h) Open book view of the V_L_/V_L_ and V_H_/V_H_ interfaces between chains 2 and 3. The residues that make contacts are shown as gray Cα spheres. (i) Molecular details of the contacts between chains 2 and 3 at (i) the V_L_/V_L_ interface and (ii) the V_H_/V_H_ interface. Potential hydrogen bonds (<3.5 Å) are shown as dashed lines.

Attempts to crystallize V_L_–V_H_ 1.1 in complex with EPOR were unsuccessful, likely due to the conformational and stoichiometric heterogeneity of the large complex. However, V_L_–V_H_ 1.1 alone crystallized readily in space group P2_1_ and we were able to solve the structure at 2.06 Å resolution with an *R*_free_ of 21.2%. The asymmetric unit contained four V_L_–V_H_ chains, and superposition revealed definitive linkages between the V_L_ and V_H_ domains (<15 Å apart) and showed that each presents a distinct conformation owing to the flexible linker (Figure 9a). The 4 chains within the asymmetric unit form 2 Dbs (chains 1/2 and chains 3/4) held together by 2 conventional V_L_/V_H_ interfaces in each, and the two Dbs pack with each other through unconventional V_L_/V_L_ and V_H_/V_H_ interfaces (between chains 2/3) to form a Tb with four antigen‐binding sites arranged asymmetrically ~59–68 Å apart (Figure 9b). Superposition of each of the four binding sites with the functional Fab binding site (described below) revealed that they all share near identical conformations and are exposed to the solvent (Figure 9c). Thus, all four sites of the Tb appear to be capable of EPOR engagement.

In addition to revealing the orientations of the four binding sites, the structure also revealed unusual packing interactions that resulted in an asymmetric Tb. Notably, no previous structure of a Tb exists, but a symmetric Tb structure has been modeled based on a symmetric triabody structure (Pei et al., 1997). In these symmetric models, conventional V_L_/V_H_ interfaces hold the oligomers together and there are no unconventional V_L_/V_L_ or V_H_/V_H_ interfaces, as seen in our structure (Figure 9b). Thus, our structure provides a unique opportunity to explore the interfaces that work together to assemble an asymmetric Tb, and we examined these in detail.

The asymmetric unit contains four V_L_/V_H_ interfaces—two in each Db—and these are all essentially identical to each other and to the V_L_/V_H_ interface in the Fab 1.1 structure (described below). To evaluate the molecular determinants for Db assembly, we superposed the V_L_/V_H_ interface observed in the Db with that of the 4D5 Fv (Eigenbrot et al., 1993), which forms spontaneously due to high‐affinity interactions between the V_L_ and V_H_ domains (Figure 4d). Analysis of these interfaces revealed that they share a similar core dominated by hydrophobic interactions with precise complementarity. Inspection of representative interfaces between the V_L_ and V_H_ domains of chains 1 and 2 revealed a total buried surface area of 1572 Å^2^ (Elliott et al., 2014) with 743 and 757 Å^2^ (Elliott et al., 2014) on the V_L_ and V_H_ side, respectively (Figure 9e). At the center of the interface, there is a hydrogen bond between the hydroxyl of the Tyr^36L^ sidechain and the amide of the Met^116H^ mainchain (Figure 9f(i); for clarity, residues in the light or heavy chain are denoted by an “L” or “H” superscript, respectively). This singular conserved polar contact is surrounded by aliphatic residues mainly in the framework but also in CDRs L3 and H3. Trp^118H^, Leu^50H^, and A^115H^ surround M^116H^ and bury the large aliphatic surface composed of Q^89L^, F^118L^, P^44L^, and L^46L^, which likewise surrounds and positions the Tyr^36L^ sidechain to form the hydrogen bond. Together, these residues make up an energetically favorable core of hydrophilic and hydrophobic interactions in the V_L_/V_H_ interface.

In addition to the core contacts, three hydrophobic clusters make up most of the buried surface area at the V_L_/V_H_ interface (Figure 9f(ii)). The first cluster involves Tyr^103H^, which makes numerous van der Waals contacts to Ala^43L^ and Pro^44L^. The second cluster involves van der Waals contacts made by Trp^52H^ with Phe^118L^, Ile^116L^, and Leu^115L^. This is further supported by Leu^50H^, which makes van der Waals contacts with Pro^44L^ and Phe^118L^ that bridge the first and second clusters. The third cluster involves van der Waals contacts made by Tyr^109H^ with Tyr^49L^, Leu^46L^, and Tyr^55L^. This is further supported by Ala^115H^, which makes van der Waals contacts with Tyr^55L^ and Leu^46L^. Thus, the formation of the Db is dictated predominantly by hydrophobic interactions nearly identical to those involved in the highly favorable association of the 4D5 Fv, and this likely represents the first step in asymmetric Tb formation.

Furthermore, each Db forms non‐covalent dimers with the other Db in the Tb (i.e., chains 2/3 and chains 1/4) through unconventional V_L_/V_L_ and V_H_/V_H_ interfaces (Figure 9g). Inspection of representative V_L_/V_L_ and V_H_/V_H_ interfaces of chains 2 and 3 revealed buried surface areas of 442 and 299 Å^2^ (Elliott et al., 2014), respectively (Figure 9h). Thus, the Tb quaternary structure is dominated by the light chain self‐association that combines the two Dbs into the Tb complex. The composite interface results in a combined total buried surface area of 759 Å^2^ (Elliott et al., 2014), but unlike the hydrophobic V_L_/V_H_ interactions, the V_L_/V_L_ and V_H_/V_H_ interactions are largely mediated by polar contacts (Figure 9i). Analysis of the V_L_/V_L_ interface revealed an extensive and largely symmetric hydrogen bond network between β‐strand 1b of each light chain — including residues Ser^9L^, Ser^10L^, Leu^11L^, and Ser^12L^ — and further supported by hydrogen bonds and van der Waals contacts made by Ala^107L^ and Asp^17L^ with Ser^7L^ (Figure 9i(i)). These interactions form a contiguous anti‐parallel β‐sheet across the homodimerized V_L_ domains. In contrast, the V_H_/V_H_ interactions are sparse and less symmetrically aligned, with Tyr^87H^ and Arg^19H^ of chain 2 making van der Waals contacts with Gln^13H^ and Leu^11H^ of chain 3. Reciprocally, Arg^19H^ of chain 3 only makes contact to Leu^11H^ (Figure 9i(ii)). Since these interfaces are mainly supported by a hydrogen bond network rather than the hydrophobic exclusion of water, it is unclear how stable these interactions are in aqueous solution. Further studies will be required to determine the conditions necessary for Tb formation and to what extent the Tb exists in solution relative to the Db, but nonetheless, the existence of a tetramer in the crystal structure is consistent with the strong agonist activity of V_L_–V_H_ 1.1 in the UT‐7/Epo cell proliferation assay (Figure 8b) and in the mouse model (Figure 8c), given that only the tetramer and higher order oligomers of the V_L_–V_H_‐Fc fusion were active in these assays (Figures 6b and 7c).

### 2.8 Crystal structure of a Fab:EPOR complex

**FIGURE 10:**
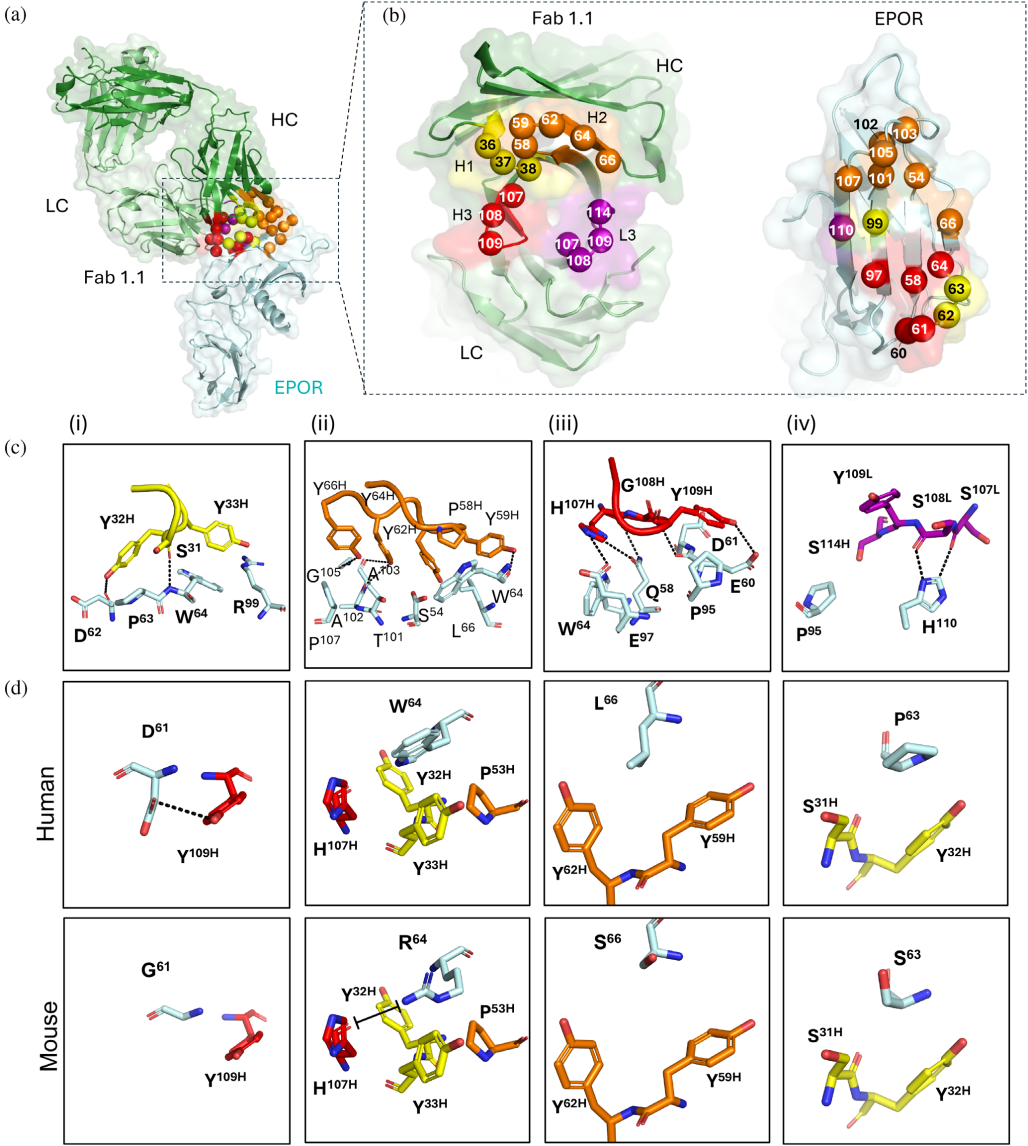
Molecular recognition of EPOR by Fab 1.1. (A) Crystal structure of Fab 1.1 bound to the EPOR ECD. The heavy chain, light chain, and EPOR are colored dark green, light green, or cyan, respectively. Contact residues from the diversified CDRs are shown as Cα spheres colored as follows: L3 (purple), H1 (yellow), H2 (orange), H3 (red). (b) Open book view of the contact surfaces of Fab 1.1 (left) and EPOR (right). Contact residues (<4.0 Å) are shown as Cα spheres numbered according to the IMGT nomenclature for the Fab and the PDB deposition for EPOR. Fab residues are colored as in (a) and EPOR residues are colored according to the CDR with which they make the most contacts. (c) Molecular details of the contacts between EPOR and CDRs (i) H1, (ii) H2, (iii) H3, and (iv) L3. Hydrogen bonds are shown as dashed lines. (d) Residues that differ between human (top) and mouse (bottom) EPOR, which are likely to contribute to the lack of Fab 1.1 binding to mEPOR. Contacts are shown between Fab CDRs and the following EPOR residues: (i) D61G, (ii) W64R, (iii) L66S, (iv) P63S.

To elucidate the structure of the interface between EPOR and the agonist Ab, we produced variant 1.1 in the monovalent Fab format, which is more amenable to crystallization. The Fab:EPOR complex crystallized in the P2_1_2_1_2_1_ space group, and we solved the structure at 2.1 Å resolution with an Rfree of 23.7% (Figure 10a). Binding of the Fab to domain 1 of EPOR results in the burial of 1009 and 1012 Å^2^ of surface area, respectively (Figure 10b). The buried surface area on the Fab was predominantly from the heavy chain (63.2%) and the various CDRs contributed the following percentages: H1 (15.4%), H2 (26.4%), H3 (21.5%), L1 (9.6%), L3 (19.7%). High affinity and specificity were mediated by a dense network of hydrophobic and polar interactions distributed across multiple CDRs (Figure 10c).

CDR‐H1 (Figure 10c(i)) uses Ser^31H^, Tyr^32H^, and Tyr^33H^ to make van der Waals contacts with EPOR residues Asp^62^, Pro^63^, Trp^64^, and Arg^99^. The sidechain of Tyr^32H^ makes a hydrogen bond to the carbonyl of Asp^62^, while the carbonyl of Ser^31H^ makes a hydrogen bond to the amine of Trp^64^. CDR‐H2 (Figure 10c(ii)) uses Pro^58H^, Tyr^59H^, Tyr^62H^, Tyr^64H^, and Tyr^66H^ to make van der Waals contacts with EPOR residues Ser^54^, Trp^64^, Leu^66^, Thr^101^, Ala^102^, Ala^103^, Gly^105^, and Pro^107^. The sidechains of Tyr^66H^ and Tyr^64H^ hydrogen bond to the carbonyl of Gly^105^ or either Ala^103^ or Ala^102^, respectively, and the sidechain of Tyr^59H^ hydrogen bonds to the carbonyl of Trp^64^. CDR‐H3 (Figure 10c(iii)) uses His^107H^, Gly^108H^, and Tyr^109H^ to make van der Waals contacts with EPOR residues Gln^58^, Glu^60^, Asp (Afonine et al., 2012), Trp^64^, Pro^95^, and Glu^97^. The carbonyl of His^107H^ hydrogen bonds to the sidechain of Gln^58^, and in different rotamers, the sidechain of His^107H^ hydrogen bonds to either the carboxyl of Gln^58^ or to the sidechain of Glu^97^. The amine of Gly^108H^ hydrogen bonds to the carbonyl of Glu^60^ and the sidechain of Try^109H^ hydrogen bonds to the sidechain of Glu^60^. CDR‐L3 (Figure 10c(iv)) uses Ser^107L^, Ser^108L^, Tyr^109L^, and Ser^114^ to make van der Waals contacts with Pro^95^ and His^110^. The carbonyl of Ser^107H^ hydrogen bonds to one rotamer of the His^110^ sidechain, while the carbonyl of Ser^108H^ hydrogen bonds to the other. Collectively, this extensive and intricate network of interactions is consistent with the high affinity between the agonist Ab and human EPOR (Figure 4a).

The structure also explained the lack of binding to mEPOR (Figure 3). Compared to human, a mEPOR complex model differs at five positions in the epitope for Fab 1.1, and all but one of these differences (R99Q) reduce contacts in the interface (Figure 10d). The D61G difference removes a hydrogen bond in the interface, and the P63S, W64R, and L66S differences reduce van der Waals contacts made primarily with Tyr residues in Fab 1.1. The W64R difference also causes close contact with His^99H^ of CDR‐H3, which may cause a charge repulsion. In sum, these differences eliminate favorable interactions and create potentially unfavorable interactions between Fab 1.1 and mEPOR.

**FIGURE 11:**
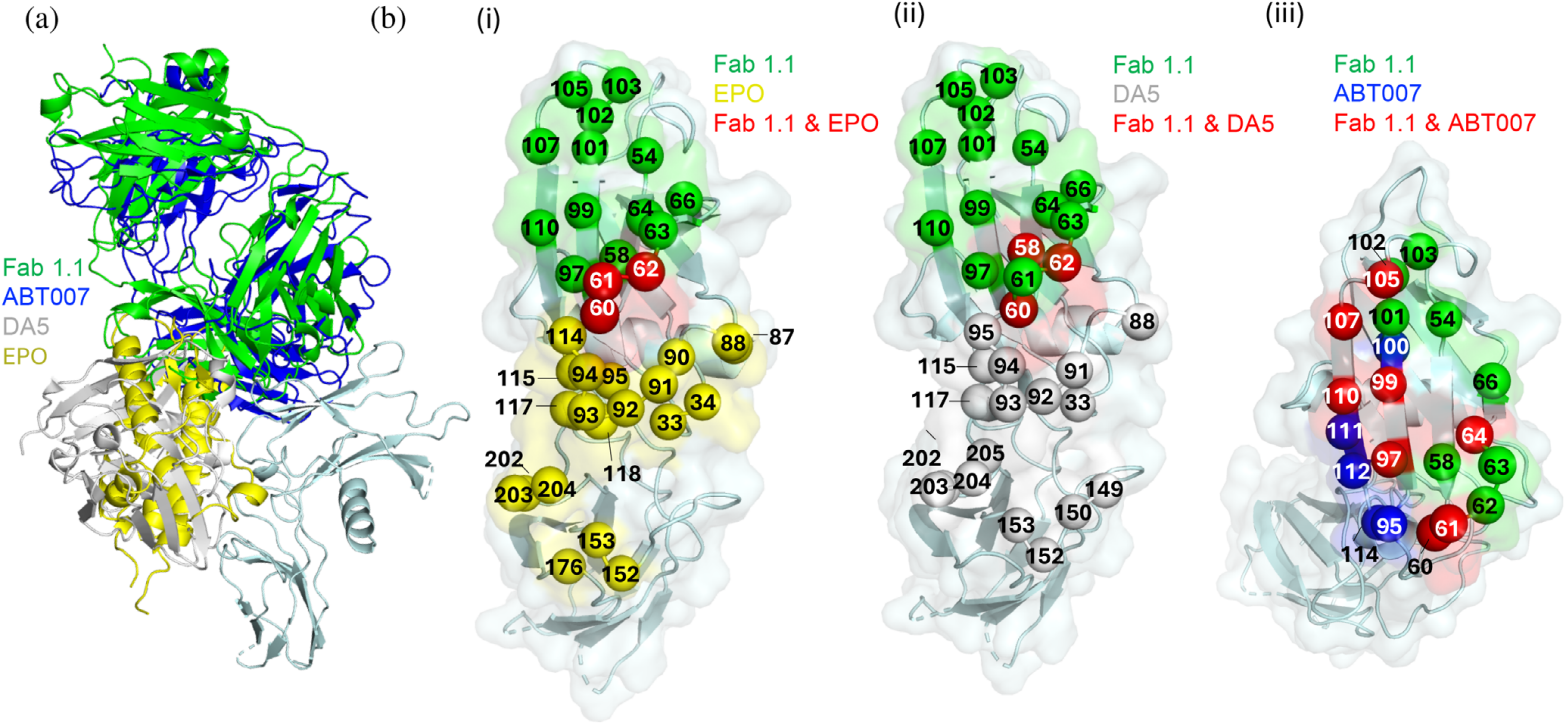
Structural comparison of putative agonists of EPOR. (A) Superposition of the Fab from the ABT007/EPOR complex (blue, PDB entry 2JIX), the scFv from the Db DA5/EPOR complex (gray, PDB entry 4Y5Y), and EPO from the EPO/EPOR site 1 complex (yellow, PDB entry 1EER) onto the structure of Fab 1.1 (green) bound to EPOR (cyan). Each complex is aligned on domain 1 of EPOR, and for clarity, only the EPOR bound to Fab 1.1 is shown as a space‐filled surface. (B) Structural contact residues (<4.0 Å) of EPOR to Fab 1.1 and (i) EPO, (ii) Db DA5, or (iii) Fab ABT007. Residues are numbered according to PDB deposition and are shown as Cα spheres. Residues contacting Fab 1.1 only are colored green, whereas residues contacting EPO, Db DA5, or Fab ABT007 only are colored yellow, gray, or blue, respectively. Residues that contact Fab 1.1 and a second ligand are colored red.

For comparison with other EPOR agonists, we superposed the Fab‐1.1:EPOR complex with structures of EPO, Db DA5, and Fab ABT007 in complex with EPOR (Figure 11a). The Fab 1.1 epitope exhibited minimal overlap with EPO site 1, with only three contacts in common (Figure 11b(i)), but the overlap was sufficient to cause steric clash between the Fab and EPO, thus explaining the competition observed in binding assays (Figure 4e). The Fab 1.1 epitope also exhibited minimal overlap with that of Db DA5, which also shared only three common contacts (Figure 11b(ii)). Instead, the Db DA5 epitope — and those of two other putative Db agonists — overlapped well with EPO site 1 (Figure S5). However, Fab 1.1 binds EPOR in a manner very similar to Fab ABT007 (Figure 11a), as these two Fabs share 8 contact residues on EPOR (Figure 11b(iii)).

Notably, and similar to our study, ABT007 is the only other putative agonist Ab that has been shown to exhibit significant activity *in vivo* using mice harboring human EPOR. However, the ABT007 agonist was produced with the IgG2 isotype that has a propensity to form disulfide‐linked IgG2 dimers with four antigen‐binding sites (Yoo et al., 2003). When we tested ABT007 and our Abs 1.1 and 1.2 in the bivalent IgG1 format, we observed no agonist activity in UT‐7/Epo cell proliferation assays (Figure S6). Taken together, these results showed that our Ab 1.1 and ABT007 recognize similar epitopes on EPOR that are distinct from the sites recognized by EPO, and in both cases, the bivalent IgG1 format is insufficient to induce agonism, but agonism is achieved with tetravalent or higher valency formats.

### 2.9 Structural model for EPOR agonism by the asymmetric Tb

The molecular mechanism by which EPOR activates the JAK:STAT signaling pathway has been extensively studied (Arcasoy & Jiang, 2005; Livnah et al., 1999). EPOR appears to exist as a preformed but inactive dimer on the cell surface (Livnah et al., 1999; Remy et al., 1999). Bivalent binding of EPO through asymmetric sites 1 and 2 seems to reconfigure the EPOR dimer into an asymmetric, active conformation. Critically, the two asymmetric EPORs must be reconfigured with precise distances and angles of engagement to trigger allosteric recruitment of the transmembrane and intracellular domains to induce transphosphorylation of associated JAK proteins.

**FIGURE 12:**
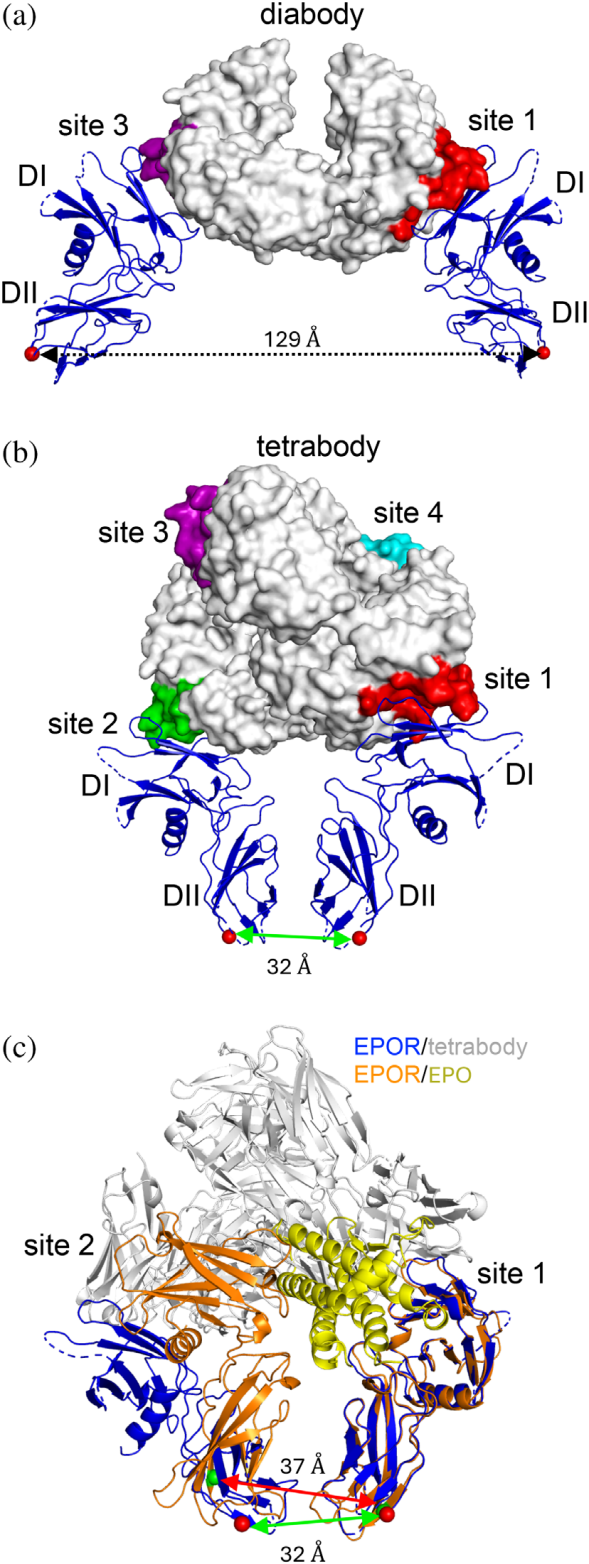
Structural model of EPOR agonism by an asymmetric Tb. (a, b) Superposition of the Fab‐1.1/EPOR complex onto (a) binding sites 1 and 3 of a single Db 1.1 or (b) binding sites 1 and 2 of the asymmetric Tb 1.1. The Ab framework is shown as a white surface with antigen‐binding sites colored as follows: 1 (red), 2 (green), 3 (purple), and 4 (cyan). The two EPORs are shown as blue ribbons, and the C‐termini are shown as red Cα spheres with the distance between them demarcated with an arrow. (c) Superposition of the EPOR dimer (blue) recruited by sites 1 and 2 of the Tb (gray) with the EPOR dimer (orange) recruited by sites 1 and 2 of EPO (yellow). The C‐termini of the two EPORs recruited by the Tb or EPO are shown as red or green Cα spheres, respectively, and the distances between them are demarcated by arrows.

With structural modeling, we assessed whether the exquisite geometry and distance requirements of an active EPOR dimer could be satisfied by the Db or Tb formats of V_L_–V_H_ 1.1. Each V_L_–V_H_ 1.1 asymmetric unit contains two distinct V_L_/V_H_‐associated V_L_‐V_H_ chain dimers that effectively represent Dbs. Superposition of the Fab‐1.1:EPOR complex structure onto one of these Dbs positioned the EPOR C‐termini too far apart (129 Å) to facilitate close recruitment of the intracellular domains (Figure 12a). Interestingly, superposition of the Fab‐1.1:EPOR complex onto the 6 possible pairs of sites contained within the Tb revealed that, due to the asymmetry of the Tb, the recruited EPOR dimers differed greatly in the proximity of their C‐termini (Figure S7a,b). Notably, the engagement mode involving sites 1 and 2 of the Tb positioned the C‐termini of the EPORs in close proximity (32 Å) (Figure 12b). Superposition of the site 1 EPOR of this Tb‐mediated dimer onto the site 1 of the EPO:(EPOR)2 complex revealed a striking conservation of the topologies of the two asymmetric EPOR dimers (Figure 12c), and remarkably, the distance between the C‐termini of the natural dimer (37 Å) was virtually identical to that induced by the Tb (32 Å). These observations suggest strongly that this engagement mode represents the critical asymmetric EPOR dimer responsible for the strong activity of our synthetic agonist.

As an independent means to verify that Tb 1.1 can position EPOR monomers in close proximity, we used a Protein‐fragment Complementation Assay (PCA) with the *Gaussia princeps* luciferase (Gluc) as a reporter. This assay is similar to a PCA that was used to assess distances between the juxtamembrane regions in an EPOR dimer induced by EPO (Remy et al., 1999; Remy & Michnick, 2006). The principle of this reporter is that the C‐termini of two interacting proteins must be in close enough proximity to permit complementary C‐terminal fragments of Gluc to fold and reconstitute its activity. The Gluc PCA fragments were fused to the C‐termini of the EPOR transmembrane domains via five‐residue linkers and were thus on the cytosolic side of the plasma membrane. Assuming peptide bond lengths of 4 Å, the two linkers define a distance limit for folding of Gluc to ~40 Å, which is similar to the transmembrane C‐terminal distance separation for the active EPOR homodimer. The EPOR Gluc PCA reporters showed similar potencies for cells treated with EPO or Db‐Fc_mix_ 1.1 (EC_50_ = 0.2 or 0.6 nM, respectively), and ~1.5‐fold greater maximal activity for the Ab (Figure S7c), which is consistent with the even closer proximity of the EPOR homodimer transmembrane C‐termini predicted to be induced by Tb‐Fc_2_ present in the Db‐Fc_mix_ compared to that induced by EPO.

Given the tetravalency of the Tb, we explored whether higher valency engagement modes beyond the active EPOR dimer may be possible. The addition of a modeled EPOR complex onto either site 3 or 4 of the Tb revealed that recruitment of a third EPOR could further stabilize engagement of the signaling EPOR dimer recruited by sites 1 and 2 (Figure S7d). Based on the crystallographic Tb geometry, a fourth engagement to EPOR on the same surface plane does not seem possible. Together with our *in vitro* and *in vivo* functional results, these structural models support a mechanistic hypothesis in which the asymmetric Tb scaffold engages pre‐dimerized EPOR molecules at sites 1 and 2 to induce a signaling‐competent, asymmetric EPOR dimer very similar to the active conformation induced by EPO itself. Moreover, this dimer may be further stabilized through avid interactions involving a third EPOR, contributing to the exceptional potency and durability of hematopoietic responses observed with this format.

## 3 DISCUSSION

Since the landmark approval of rEPO as an ESA for treatment of anemia in 1989 (D'Andrea et al., 1989), intensive efforts have sought to develop alternatives with better manufacturability, stability, half‐life, and safety (Sinclair, 2013). Nonetheless, more than three decades later, all clinically approved EPOR agonists are derivatives of EPO, with the only practical advance being extended half‐life via hyperglycosylation (darbepoeitin) (Egrie & Browne, 2001) or pegylation (mircera) (Jarsch et al., 2008). Furthermore, the promiscuity of EPO for alternative receptors has been associated with pleiotropic effects, including a potential risk of tumorigenesis (Debeljak et al., 2014). Indeed, mircera is explicitly contraindicated for patients with chemotherapy‐induced anemia (https://www.mircera.global), limiting EPO‐based ESAs to only a subset of the patients that could otherwise benefit.

Given their ideal characteristics for development as therapeutic biologics — including stability, manufacturability, and specificity — intensive efforts have been made to develop Ab agonists of EPOR. However, despite exhaustive efforts, no candidate Ab‐based ESA has been advanced into clinical trials (Elliott et al., 1996; Liu et al., 2007; Moraga et al., 2015). We noted that all efforts to date have utilized bivalent Abs, and we wondered whether higher valency Abs may be able to bind EPOR and induce an active state comparable to that induced by EPO.

Here, we elucidated fundamental relationships between the epitopes and oligomeric states of Abs, and their ability to stimulate EPOR. Our findings illuminate the inherent complexities that likely limited previous therapeutic development of Ab‐based EPOR agonists and provide a path toward the development of Ab‐based ESAs that can address many of the limitations of EPO‐based ESAs.

We report two fundamental findings. First, effective Ab‐based agonists of EPOR do not bind to the EPO‐binding sites on EPOR, but rather, to an epitope “on top” of the receptor. Binding at this novel site appears to enable Abs to bring EPOR dimers in closer proximity than is possible through binding to the canonical EPO‐binding sites. Second, for Ab preparations that exhibited strong agonism of EPOR, the activity did not reside in the predominant bivalent form, but rather, in higher‐order oligomers. The minimum active form was tetravalent, and structural studies suggest that this active form exists as an asymmetric Tb. Moreover, structural modeling suggests that the asymmetric nature of the Tb is essential for its ability to recruit an asymmetric dimer of EPOR that closely resembles the active dimer recruited by EPO and positions the juxtamembrane regions in close proximity with a conformation virtually identical to that induced by EPO. Although only one of six possible EPOR pairs formed by the Tb appears to mimic the active dimer, pre‐assembly of the EPOR dimer on the cell surface may favor binding to these two sites, as their geometry enables cooperative binding. Moreover, trivalent engagement may further stabilize the signaling complex through avidity. Regardless of the exact molecular mechanism, we showed conclusively that the asymmetric Tb is an extremely effective agonist of EPOR both *in vitro* and *in vivo*. Indeed, in mice bearing the human EPOR, the potency of the Tb matched — and the efficacy and durability exceeded — those of darbepoetin, the current dominant therapeutic EPOR agonist.

Despite these promising findings, significant challenges remain for the translation of the Tb format into a viable therapeutic. The active Tb in our protein preparations was serendipitous, and it was a minor component with the inactive Db dominating the expression products. For drug manufacturing, the active species should ideally be the major product of recombinant protein expression, and it should be easily purifiable from minor products and contaminants. Intriguingly, in variant 1.3, changes in the CDRs at positions that do not make intermolecular contacts between V_L_–V_H_ chains were able to significantly increase the proportion of the protein preparation in the active tetrameric conformation. With the high‐resolution crystal structure of the asymmetric Tb in hand, it should be possible to optimize the V_L_/V_L_ and V_H_/V_H_ intermolecular contacts that appear to stabilize the Tb formed by the two Dbs in the asymmetric unit. In addition, the length and composition of the linker between the V_L_ and V_H_ domains can be manipulated to favor particular oligomeric states.

Alternative formats for tetravalent Abs may also prove successful for agonism of EPOR. Notably, we have recently shown that Db‐Fc‐Db and Db‐Fc‐Fab tetravalent formats can be used to stimulate regenerative FZD/LRP signaling, and one such agonist has been manufactured successfully and is currently in a phase 2b/3 clinical trial for diabetic macular edema (NCT06571045). Efforts in these directions are underway, and we believe that an effective Ab‐based agonist of EPOR will soon be available as a therapeutic for anemia.

In conclusion, our findings identify and validate a novel mechanism of EPOR activation by Ab‐based agonists, distinct from EPO and its derivatives. By targeting a unique epitope and leveraging asymmetric tetravalent Abs, we have developed synthetic agonists that exceed the performance of existing ESAs. These insights pave the way for safer and more effective EPOR‐targeted therapies, and they also offer a general strategy for engineering agonist Abs for other therapeutically relevant receptors that rely on clustering for activation.

## 4 METHODS

### 4.1 Phage‐displayed library construction

To construct a naïve, synthetic Db library, we modified a phagemid vector encoding a human framework scFv (Nelson & Sidhu, 2012; Van Deventer et al., 2015). In our design, the C‐terminus of the light‐chain variable (V_L_) domain is linked to the N‐terminus of the heavy‐chain variable (V_H_) domain by a Gly_5_ linker, producing an open reading frame encoding for a Db linked to the C‐terminal domain of the M13 gene‐3 minor coat protein with a modified IgG hinge sequence. To enable facile reformatting into protein expression vectors, we designed the phagemid vector with unique restriction sites flanking the Db‐encoding gene. The phagemid was used to construct a Db library in which 4 of the 6 CDRs were diversified with a tailored strategy. The three heavy chain CDRs and CDR‐L3 were diversified as described previously for a Fab library (Persson et al., 2013), but with reduced length diversity for CDR‐H3. The library contained 4.2 × 10^9^ unique clones, and DNA sequencing of 74 clones revealed the incorporation of diversity in approximately 70% of the population within each CDR, with template sequence retained for the remainder. Overall, 12%, 11%, and 50% of the library members contained diversity in two, three, or four CDRs, respectively.

For sub‐libraries, oligonucleotide‐directed combinatorial mutagenesis was used to simultaneously diversify positions in CDRs H1, H2, H3, and L3 in Db 1 as described (Persson et al., 2013), using degenerate oligonucleotides in which codons were replaced with a mixture that retained at least 49% of the wildtype base, as described (Gallo et al., 2021). The mutagenesis reaction was electroporated into *E. coli* SS320 to construct a library containing >10^9^ variants. The same methodology was applied to CDRs L3, H1, H2, and H3 of Db 1.1 to derive Db variants 1.2 and 1.3.

### 4.2 Phage display selections

Phage pools representing libraries were cycled through rounds of binding selection with antigen coated on 96‐well Maxisorp Immunoplates (Nunc, Rochester, NY), as described (Fellouse et al., 2007; Sidhu et al., 2004). After four rounds of selection, phage were produced from individual clones grown in a 96‐well format, and the culture supernatants were used in phage ELISAs to detect specific binding clones. Clones that bound to antigen but not to BSA (Sigma‐Aldrich, St. Louis, MO) were subjected to DNA sequence analysis.

### 4.3 Expression and purification of Ab proteins

Db‐Fc proteins were expressed in mammalian cells using the Expi293 transient expression system (ThermoFisher). Briefly, plasmids (pSCSTa) designed to express V_L_–V_H_ or V_L_–V_H_‐Fc chains were co‐transfected into Expi293 cells according to the manufacturer's instructions. After 5 days, cell culture medium was harvested and applied to an rProtein‐A affinity column (Cytiva GE17‐1279‐03). Ab protein was eluted with IgG elution buffer (Pierce) and immediately neutralized with 1 M Tris–NaCl pH 8. Fractions containing eluted Ab protein were combined, concentrated, and dialyzed into PBS, pH 7.4. Ab protein was characterized for purity by SDS‐PAGE, and concentration was determined by spectrophotometry at an absorbance wavelength of 280 nm.

### 4.4 Analytical SEC

Samples were diluted to 1 mg/mL in PBS (ThermoFisher 10010031). Each sample (50 μL) was centrifuged at 14,000 rpm for 10 min to remove precipitates, and the supernatant was transferred into an HPLC glass vial (Sigma‐Aldrich 29122‐U). The main column used was the TSKgel G3000SW_xL_, 7.8 mm ID × 30 cm (Sigma‐Aldrich 808541) and the guard column was TSKgel G3000SW_xL_: 6 mm ID × 4 cm (Sigma‐Aldrich 808543). Columns were equilibrated with filtered and de‐gassed PBS for 30 min at a flow rate of 0.5 mL/min. The sample (20 μL) was injected at a flow rate of 0.5 mL/min with a run time of 40 min.

### 4.5 Preparative SEC

Samples were diluted to 5–10 mg/mL in PBS (ThermoFisher 10010031) in a final volume of 1–2 mL and filtered using a 0.45‐μm syringe filter. Samples were centrifuged and the supernatant (40 μL) was loaded into an HPLC glass vial (Sigma‐Aldrich 29122‐U). Samples (1–2 mL) were run for 90 min at a flow rate of 1.0 mL/min with a maximum pressure of 2 MPa on a Superdex 75 increase Hiscale column (GE Healthcare 29321907) that was equilibrated with de‐gassed PBS for 90 min. Collection was performed at 1 fraction per minute.

### 4.6 ELISAs

A 384‐well maxisorp plate was coated overnight at 4°C with 10 μL/well of 2 μg/mL His‐EPOR (Sino Biological 10707‐H08H) in PBS. A control plate was also coated with 0.5% BSA in PBS. Both plates were blocked with 0.5% BSA in PBS (60 μL/well) for 1 h at room temperature. Plates were washed twice with PBST (50 μL/well). Db‐Fc solutions were added (25 μL/well), the plates were incubated at room temperature for 1 h, washed six times with PBST buffer (50 μL/well), and incubated for 30 min at room temperature with human anti‐Fc‐HRP (Jackson Immunoresearch 109‐035‐098) diluted 1:5000 in PBST (25 μL/well). Plates were washed six times with PBST (50 μL/well) and developed with TMB reagent (KPL‐Mandel KP‐50‐76‐03, 25 μL/well) according to the manufacturer's instructions. The reaction was quenched with 1 M phosphoric acid (25 μL/well) and absorbance was read at 450 nm.

### 4.7 Biolayer interferometry

Binding kinetics (*k*_on_ and *k*_off_) and dissociation constants (*K*_D_) of Abs for His‐EPOR were determined by BLI using an Octet HTX instrument (ForteBio) at 1000 rpm and 25°C. Kinetic assays were performed by capturing the Abs (5 μg/mL) to achieve a binding response of 0.4–0.6 nm using anti‐human Fc Octet biosensors. Non‐occupied sites were quenched by dipping the biosensors in wells containing 50 μg/mL of human IgG1 Fc protein for 600 s. Abs were diluted with assay buffer (PBS, 0.05% Tween 20), and a non‐binding Ab was used as a negative control. The loaded sensors were dipped in wells containing the assay buffer for the baseline, and then dipped for 600 s into wells containing 3‐fold serial dilutions of His‐EPOR starting at 100 nM for the association, and subsequently, were transferred back into the baseline wells for 600 s for dissociation. Binding response data were corrected by subtraction of response from a reference and were fitted with a 1:1 binding model using ForteBio Octet Systems software 9.0.

### 4.8 Competition assays

Binding kinetics (*k*_on_ and *k*_off_) and dissociation constants (*K*_D_) of Abs binding to the His‐EPOR in the presence of competing Abs were determined by BLI using an Octet HTX instrument (ForteBio) at 1000 rpm and 25°C. Assays were performed by capturing His‐EPOR (5 μg/mL) to achieve a binding response of 0.4–0.6 nm using anti‐His Octet biosensors. Loaded tips were dipped in 200 nM Ab samples in assay buffer (PBS, 0.05% Tween 20) for 600 s and then in 100 nM samples of the second Ab. Readings were taken at 20 s using ForteBio Octet Systems software 9.0.

### 4.9 Flow cytometry

Sub‐confluent cells were collected and washed twice in 10 mL of ice‐cold FACS buffer (PBS, 0.5% BSA, 0.05% sodium azide). Cells were kept cold to reduce receptor trafficking. Each FACS assay was performed with 2.5 × 10^5^ cells, so the total cell number was adjusted based on the experiment accordingly. Cells were pelleted by centrifugation at 4°C and resuspended in ice‐cold FACS buffer to a concentration of 2.5 × 10^6^ cells/mL, and 100 μL aliquots were pipetted into each well of a 96‐well plate. To each sample, 1 μg of Fc Ab (purified rat anti‐mouse CD16/CD32 mouse BD Fc block, BD Biosciences 553142) was added and incubated at 4°C for 15 min. The corresponding Ab was added (200 nM) to each sample. The Abs and cells were mixed gently by shaking for 1 min at 4°C and incubated at 4°C for 45 min. Cells were pelleted by centrifuging at 500g for 5 min at 4°C and washed with 200 μL of ice‐cold FACS buffer. After three washes and aspiration, cells were resuspended in 100 μL secondary Ab solution (ice cold FACS buffer with anti‐human Fc AlexaFluor‐488, 1:500 dilution, Jackson ImmunoResearch 109‐545‐008) and incubated on ice for 20 min. Cells were pelleted and washed three times with 200 μL of ice‐cold FACS buffer.

### 4.10 UT‐7/Epo cell proliferation assay

UT7/Epo cells (Kim et al., 2017), a generous gift from Dr. Vijay Sankaran at Harvard Medical School, were cultured in DMEM medium (ThermoFisher 11995‐073) with 10% FBS and 2 U/mL EPO (Cell Sciences CRE600D) and passaged every 2–3 days. To measure proliferation, cells were washed twice with PBS, resuspended in DMEM medium without FBS and EPO (starvation medium), incubated overnight, and seeded at a density of 10,000 cells/well in 90 μL of starvation medium in 96‐well plates. Each treatment was performed in triplicate with EPO and Abs stocks prepared in starvation media at 1 μM. Three‐fold serial dilutions were prepared for a total of 8 concentrations. Each stock (10 μL) was added to 90 μL of cells. Cells were cultured for 3 days in these mixes or in starvation media as a negative control. Cell‐Titer Glo (Promega G9242) was used according to the manufacturer's instructions.

### 4.11 TF1 cell proliferation assay

TF1 cells (ATCC CRL‐2003) were cultured similar to UT7/Epo cells with the following exceptions. Cells were grown in RPMI medium (Gibco A10491‐01) with 10% FBS and 2 ng/mL GM‐CSF (R&D Systems 7954‐GM‐010) and were passaged every 2–3 days. Starvation media was RPMI without FBS and GM‐CSF. For treatments, instead of GM‐CSF, EPO or Ab was added at the same serial concentrations as UT7/Epo cell assays.

### 4.12 Immunoblotting

UT7/Epo cells were incubated overnight in starvation medium and treated with EPO or Ab for 5 min. Cells were harvested by centrifugation at 500g for 5 min at 4°C, followed by a wash with ice‐cold PBS. Cells were lysed in NP‐40 lysis buffer (50 mM Tris–HCl, pH 7.4, 150 mM NaCl, 1% NP‐40, 5 mM EDTA) supplemented with phosphatase (ThermoFisher Scientific A32957) and protease inhibitors (ThermoFisher Scientific A32955). Lysates were resolved by SDS‐PAGE and analyzed by immunoblotting using the following Abs: anti‐pJAK2 (Tyr1007/1008) Rabbit mAb C80C3, 3776S Cell Signalling Technologies; anti‐JAK2 Rabbit mAb D2E12, 3230S Cell Signalling Technologies; anti‐STAT5 (A‐9), sc‐74442 Santa Cruz; anti‐pSTAT5 (Tyr694) (14H2) Mouse mAb 9356, Cell Signalling Technologies; anti‐pSTAT3 (Tyr705) Mouse mAb, 9138S Cell Signalling Technologies; anti‐STAT3 (79D7) Rabbit mAb, 4904S Cell Signalling Technologies; anti‐pERK1/2 (Thr202/Tyr204), 9101 Cell Signalling Technologies; anti‐ERK1/2 (MK1), sc‐135900 Santa Cruz; anti‐pAKT (Ser473), 9271 Cell Signalling Technologies.

### 4.13 Crystallography

Crystallization of the V_L_–V_H_ 1.1 Tb was performed in sitting drops. Optimal crystallization occurred in a liquor containing 20% PEG8000 and 100 mM CHES, using micro‐seeding to improve morphology. Crystals were cryoprotected in the same liquor plus 25% ethylene glycol and were sent for diffraction at beamline 24‐ID‐E at the Northeastern Collaborative Access Team (NECAT) at the Advanced Photon Source at Argonne National Laboratory (Argonne, IL). Datasets were collected remotely from a single crystal at 100 K. Following data collection, data were indexed, integrated, and scaled with Aimless (Collaborative Computational Project N 4, 1994) within the autoPROC toolbox (Vonrhein et al., 2011). The structure was solved by molecular replacement using PHASER (Afonine et al., 2012; McCoy et al., 2007) in the PHENIX crystallography suite (Afonine et al., 2012) using four copies of a model of the Fv of Fab^S1CE^‐EPR‐1 (PDB entry 8VUI). The model was improved with multiple cycles of phenix.refine, model building with Coot (Emsley et al., 2010), and water picking within phenix.refine. TLS parameterization (Urzhumtsev et al., 2013) was also used during later stages of refinement. Crystallization of Fab 1.1 in complex with the extracellular domain of EPOR, as well as data collection, structure determination, and model refinement were described previously (Bruce et al., 2024) (PDB entry 8VUI).

### 4.14 *Gaussia princeps* luciferase protein‐fragment complementation assay

The EPOR‐Gluc PCA was composed of the extracellular and transmembrane domains of EPOR (residues 1–270) fused to one of two complementary fragments of Gluc (F [1] or F [2]) through a five‐residue GGGGS linker, thus generating EPOR(1–270)‐5aa‐GlucF [1] and EPOR(1–270)‐5aa‐GlucF [2]. The five‐residue linker was based on a previous report that with this length, complementation of the PCA fragments only occurs when EPOR agonists are bound (Remy et al., 1999). To overcome the rapid light decay from wild‐type Gluc, we used a variant (Gluc4) with 3 mutations (L30S/L40P/M43V) in F [1] (Degeling et al., 2013; Remy et al., 1999; Remy & Michnick, 2006).

Plasmids encoding for EPOR(1–270)‐5aa‐GlucF [1] and EPOR(1–270)‐5aa‐GlucF [2] were co‐transfected in a 1:1 ratio (200 ng total DNA per well) into CHO cells plated on 96‐well clear‐bottomed white microtitre plates (Corning), using ViaFect transfection reagent according to the manufacturer's instructions. Transfected cells were grown in Dulbecco's Modified Eagle's Medium (DMEM) without phenol red or FBS. Luminescence assays were performed 48 h after transfection. On the day of the screen, cells (~20,000 per well) were washed twice with PBS. Medium was exchanged for DMEM (100 μL/well) without phenol red or FBS. Serial dilutions of putative agonists were added to the medium, and samples were incubated at 37°C for 30 min. Three wells of transfected cells were left untreated to be used as negative controls. Native coelenterazine was added to the wells at a final concentration of 50 μM and samples were read on a Spark 1507001200 microtitre plate fluorescence/luminescence reader fitted with a microinjector (Tecan USA).

### 4.15 *In vivo* erythroid stimulation

All animal procedures were approved by the Institutional Animal Care and Use Committee (IACUC) at the University of Texas Southwestern Medical Center (UTSW) in Dallas, TX, and conducted in accordance with institutional and national guidelines. Male and female humanized EPOR knock‐in mice (C57BL/6 background, at least 3 months old), expressing human EPOR in place of mEPOR, were used for all *in vivo* experiments. These mice were first described by Divoky et al. (Divoky et al., 2001) and were kindly provided by Dr. Mary Hartnett (University of Utah).

Multiple independent experiments were performed using Db‐Fc variants at a dose of 1 mg/kg, administered intraperitoneally and diluted in sterile PBS. Darbepoetin alfa (Aranesp, Amgen) was included as a positive control at 0.1 mg/kg. A control Ab targeting the SARS‐CoV‐2 spike protein was used as the negative control for all comparator injections. Following treatment, blood was collected at multiple time points for up to 84 days, depending on the experimental design. Complete blood counts were obtained using a Hemavet 950 (Drew Scientific).

### 4.16 Quantification of Ab levels in serum

Serum concentrations of Abs were measured using a capture ELISA specific for human IgG Fc. Greiner high‐binding 96‐well plates were coated overnight in PBS at 4°C with 100 μL/well of AffiniPure Goat Anti‐Human IgG Fcγ fragment‐specific Ab (Jackson ImmunoResearch, 2 μg/mL). Plates were washed three times with PBST (PBS, 0.05% Tween‐20) and then blocked with 200 μL/well of 3% BSA in PBST for 1 h at room temperature. After blocking, plates were washed with PBST.

Serum samples were diluted (1:10 to 1:1000) in PBST, 3% BSA containing the appropriate amount of mouse serum to match the dilution of the test samples. Standards were prepared using purified Db‐Fc, diluted in the same buffer as the test samples, at concentrations ranging from 10 μg/mL to 1 ng/mL. Aliquots (100 μL) of each diluted sample or standard were added, plates were incubated for 1 h at room temperature, and washed three times with PBST. Detection was performed using 100 μL/well of HRP‐conjugated Goat Anti‐Human IgG Fcγ fragment‐specific Ab (Jackson ImmunoResearch), diluted 1:5000 in PBST and incubated for 1 h at room temperature. Plates were washed, and 50 μL TMB substrate (ThermoFisher) was added per well and incubated for 3 min. The reaction was stopped with 50 μL of 2 N H_2_SO_4_ per well, and absorbance was measured at 450 nm using a plate reader (BioTek Synergy HTX). Standard curves were generated using purified Db‐Fc, and sample concentrations were calculated by interpolation.

## AUTHOR CONTRIBUTIONS

**Jarrett J. Adams:** Conceptualization; supervision; writing – original draft; writing – review and editing; methodology; data curation; formal analysis. **Levi L. Blazer:** Conceptualization; investigation; methodology; formal analysis; data curation; supervision; writing – review and editing. **Jacky Chung:** Data curation; formal analysis; supervision; project administration; writing – original draft; writing – review and editing. **Minoo Karimi:** Methodology; validation; data curation; supervision; investigation. **Taylor Davidson:** Methodology; data curation; investigation; formal analysis; writing – original draft. **Heather A. Bruce:** Investigation; methodology; validation; formal analysis; data curation. **Alexander U. Singer:** Conceptualization; methodology; data curation; investigation; formal analysis; writing – original draft. **Ning Yang:** Conceptualization; methodology; data curation; investigation; formal analysis. **Lia Cardarelli:** Conceptualization; methodology; data curation; investigation; formal analysis. **Isabelle Pot:** Conceptualization; funding acquisition; supervision; project administration; writing – original draft; writing – review and editing. **Luigi Colombo:** Project administration; supervision; writing – review and editing; resources. **Lily Jun‐shen Huang:** Writing – review and editing; investigation; formal analysis; methodology. **Yue Ma:** Methodology; investigation; formal analysis. **Stephen W. Michnick:** Project administration; writing – original draft; writing – review and editing; investigation; formal analysis; conceptualization. **Orson W. Moe:** Conceptualization; methodology; data curation; investigation; formal analysis; supervision; funding acquisition; project administration; writing – original draft; writing – review and editing. **Sachdev S. Sidhu:** Conceptualization; methodology; data curation; investigation; formal analysis; supervision; funding acquisition; project administration; writing – original draft; writing – review and editing.

## Supporting information


**TABLE S1:** Supporting information.

**FIGURE S1.** Fusion protein design for a phage‐displayed Db. (a) The domain structure of the fusion protein consists of the following: secretion signal sequence, FLAG tag (red), VL domain (blue), Gly5 linker (black), VH domain (green), hinge (black), C‐terminal domain of the gene‐3 minor coat protein from M13 bacteriophage (gray). CDRs that were diversified in the library are demarcated by black lines and labeled (L3, H1, H2, H3). (b) Sequence of the fusion protein. Domains are colored as in (a), VH and VL are in bold text, and CDRs that were diversified are labeled.
**FIGURE S2:** Flow cytometry. Flow cytometry data are shown for TF‐1 cells and each Db‐Fc protein (100 nM, red) compared with secondary conjugated to Alex 488 alone (black).
**FIGURE S3:** BLI assays for Db‐Fc proteins binding to EPOR. BLI sensor traces (black) are shown for immobilized EPOR‐Fc binding to Db‐Fc (a) 1, (b) 2, (c) 1.1, (d) 1.2, and (e) 1.3. Indicated concentrations of Db‐Fc were allowed to associate for 600 s, dissociation was monitored for an additional 600 s (*x*‐axis), and the response was measured (*y*‐axis). The curves were globally fit (red) to a 1:1 binding model, and derived K_D_ values are shown above.
**FIGURE S4:** Size exclusion chromatography of Db‐Fcs 1.2. (a) A mixture of Db‐Fc 1.2 purified by protein‐A affinity chromatography was separated into four fractions. The fractionated volume corresponding to Db‐Fc molecular weight is highlighted in yellow.
**FIGURE S5:** Superposition of the Fab‐1.1:EPOR complex with EPOR in complex with EPO and Dbs. (a) EPOR is colored cyan and Fab 1.1 is colored gray. EPO bound to site 1 (PDB entry 1EER) is colored yellow. Db DA5, DA330, and DA10 (PDB entries 4Y5V, 4Y5Y, and 4Y5X, respectively) are colored, peach, magenta, or yellow, respectively.
**FIGURE S6:** UT‐7/Epo cell proliferation assays to assess EPOR agonist activity of IgG1 Abs. (a) UT‐7/Epo cell proliferation (*y*‐axis) was monitored in the presence of various concentrations (*x*‐axis) of positive control EPO (unfilled red circles), a negative isotype control Ab (unfilled black hexagons), or the following IgG1 Abs: 1.1 (filled green triangles), 1.2 (filled inverted orange triangles), ABT007 (filled cyan squares). Cell numbers and viability were assessed by the ATP/luciferase luminescence.
**FIGURE S7:** Structural models for EPOR dimers recruited by the asymmetric Tb. (a) Unproductive EPOR bivalent engagements by Tb 1.1. The Fab‐1.1/EPOR complex was superposed onto 5 of 6 binding site pairs of Tb 1.1 that lead to unproductive recruitment of EPOR dimers (>100 Å distance between C‐termini). (b) Productive EPOR bivalent engagement (32 Å distance between C‐termini). The Fab‐1.1/EPOR complex was superposed onto sites 1 and 2 of Tb 1.1. (c) Gluc PCA activity (y‐axis) reporting proximity of EPOR homodimers in response to treatment with various concentrations of EPO, Db‐Fc_mix_ 1.1, or a negative control IgG (*x*‐axis). Gluc luminescence activity can only be observed if the EPOR homodimer transmembrane domains are within ~40 Å of each other. (d) Engagement of a third EPOR through site 4 to reinforce the EPOR signaling dimer recruited through sites 1 and 2. The Fab 1.1/EPOR complex was superposed onto the Tb structure on sites 1, 2, and 4 to form a trivalent plane of engagement at a modeled cell membrane. The Tb is colored by V_L_–V_H_ chain (blue, orange, green, or cyan), and EPOR is shown as a surface (cyan).

## Data Availability

The data that support the findings of this study are openly available in PDB bank at https://www.rcsb.org/, reference number 8VUI, 9NWU.
