# Supplementary material for: An asymmetric tetrabody is a potent and efficacious agonist of the erythropoietin receptor in vitro and in vivo
Source: Protein Sci. 2025 Sep 17;34(10):e70292. doi: 10.1002/pro.70292 (PMC12442453; doi:10.1002/pro.70292)
Supplement: Supplementary file 2 — FIGURE S1. Fusion protein design for a phage‐displayed Db. (a) The domain structure of the fusion protein consists of the following: secretion signal sequence, FLAG tag (red), VL domain (blue), Gly5 linker (black), VH domain (green), hinge (black), C‐terminal domain of the gene‐3 minor coat protein from M13 bacteriophage (gray). CDRs that were diversified in the library are demarcated by black lines and labeled (L3, H1, H2, H3). (b) Sequence of the fusion protein. Domains are colored as in (a), VH and VL are in bold text, and CDRs that were diversified are labeled. FIGURE S2: Flow cytometry. Flow cytometry data are shown for TF‐1 cells and each Db‐Fc protein (100 nM, red) compared with secondary conjugated to Alex 488 alone (black). FIGURE S3: BLI assays for Db‐Fc proteins binding to EPOR. BLI sensor traces (black) are shown for immobilized EPOR‐Fc binding to Db‐Fc (a) 1, (b) 2, (c) 1.1, (d) 1.2, and (e) 1.3. Indicated concentrations of Db‐Fc were allowed to associate for 600 s, dissociation was monitored for an additional 600 s (x‐axis), and the response was measured (y‐axis). The curves were globally fit (red) to a 1:1 binding model, and derived KD values are shown above. FIGURE S4: Size exclusion chromatography of Db‐Fcs 1.2. (a) A mixture of Db‐Fc 1.2 purified by protein‐A affinity chromatography was separated into four fractions. The fractionated volume corresponding to Db‐Fc molecular weight is highlighted in yellow. FIGURE S5: Superposition of the Fab‐1.1:EPOR complex with EPOR in complex with EPO and Dbs. (a) EPOR is colored cyan and Fab 1.1 is colored gray. EPO bound to site 1 (PDB entry 1EER) is colored yellow. Db DA5, DA330, and DA10 (PDB entries 4Y5V, 4Y5Y, and 4Y5X, respectively) are colored, peach, magenta, or yellow, respectively. FIGURE S6: UT‐7/Epo cell proliferation assays to assess EPOR agonist activity of IgG1 Abs. (a) UT‐7/Epo cell proliferation (y‐axis) was monitored in the presence of various concentrations (x‐axis) of positive control EPO (un [file PRO-34-e70292-s002.pptx]

## Slide 1
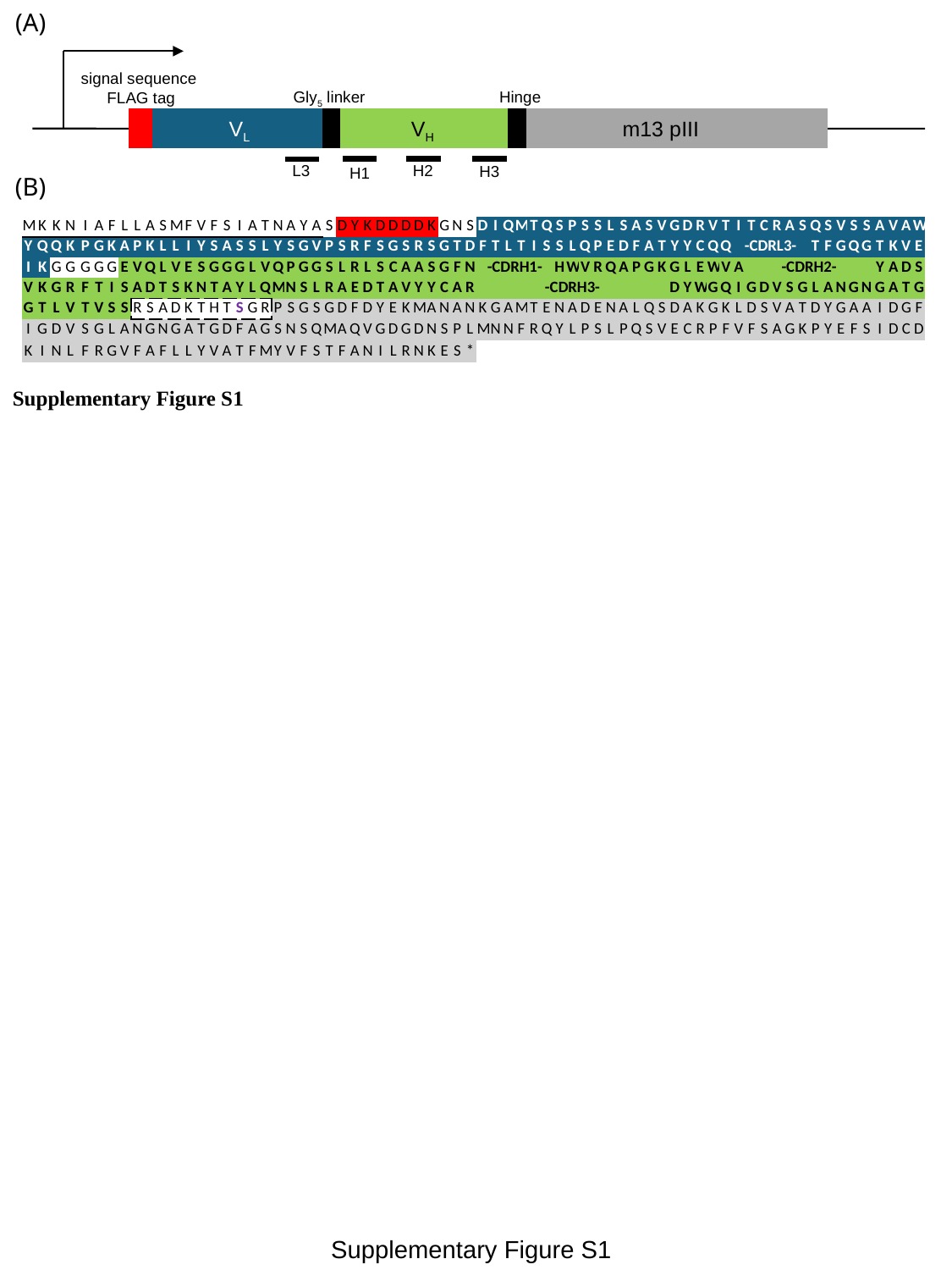

(A)
signal sequence
FLAG tag
Gly5 linker
Hinge
VL
 VH
m13 pIII
L3
H2
H3
H1
(B)
| M | K | K | N | I | A | F | L | L | A | S | M | F | V | F | S | I | A | T | N | A | Y | A | S | D | Y | K | D | D | D | D | K | G | N | S | D | I | Q | M | T | Q | S | P | S | S | L | S | A | S | V | G | D | R | V | T | I | T | C | R | A | S | Q | S | V | S | S | A | V | A | W |
| --- | --- | --- | --- | --- | --- | --- | --- | --- | --- | --- | --- | --- | --- | --- | --- | --- | --- | --- | --- | --- | --- | --- | --- | --- | --- | --- | --- | --- | --- | --- | --- | --- | --- | --- | --- | --- | --- | --- | --- | --- | --- | --- | --- | --- | --- | --- | --- | --- | --- | --- | --- | --- | --- | --- | --- | --- | --- | --- | --- | --- | --- | --- | --- | --- | --- | --- | --- | --- | --- |
| Y | Q | Q | K | P | G | K | A | P | K | L | L | I | Y | S | A | S | S | L | Y | S | G | V | P | S | R | F | S | G | S | R | S | G | T | D | F | T | L | T | I | S | S | L | Q | P | E | D | F | A | T | Y | Y | C | Q | Q | -CDRL3- | | | | | | T | F | G | Q | G | T | K | V | E |
| I | K | G | G | G | G | G | E | V | Q | L | V | E | S | G | G | G | L | V | Q | P | G | G | S | L | R | L | S | C | A | A | S | G | F | N | -CDRH1- | | | | | | H | W | V | R | Q | A | P | G | K | G | L | E | W | V | A | -CDRH2- | | | | | | | | | | Y | A | D | S |
| V | K | G | R | F | T | I | S | A | D | T | S | K | N | T | A | Y | L | Q | M | N | S | L | R | A | E | D | T | A | V | Y | Y | C | A | R | -CDRH3- | | | | | | | | | | | | | | | D | Y | W | G | Q | I | G | D | V | S | G | L | A | N | G | N | G | A | T | G |
| G | T | L | V | T | V | S | S | R | S | A | D | K | T | H | T | S | G | R | P | S | G | S | G | D | F | D | Y | E | K | M | A | N | A | N | K | G | A | M | T | E | N | A | D | E | N | A | L | Q | S | D | A | K | G | K | L | D | S | V | A | T | D | Y | G | A | A | I | D | G | F |
| I | G | D | V | S | G | L | A | N | G | N | G | A | T | G | D | F | A | G | S | N | S | Q | M | A | Q | V | G | D | G | D | N | S | P | L | M | N | N | F | R | Q | Y | L | P | S | L | P | Q | S | V | E | C | R | P | F | V | F | S | A | G | K | P | Y | E | F | S | I | D | C | D |
| K | I | N | L | F | R | G | V | F | A | F | L | L | Y | V | A | T | F | M | Y | V | F | S | T | F | A | N | I | L | R | N | K | E | S | \* | | | | | | | | | | | | | | | | | | | | | | | | | | | | | | | | | | | |
Supplementary Figure S1
Supplementary Figure S1

## Slide 2
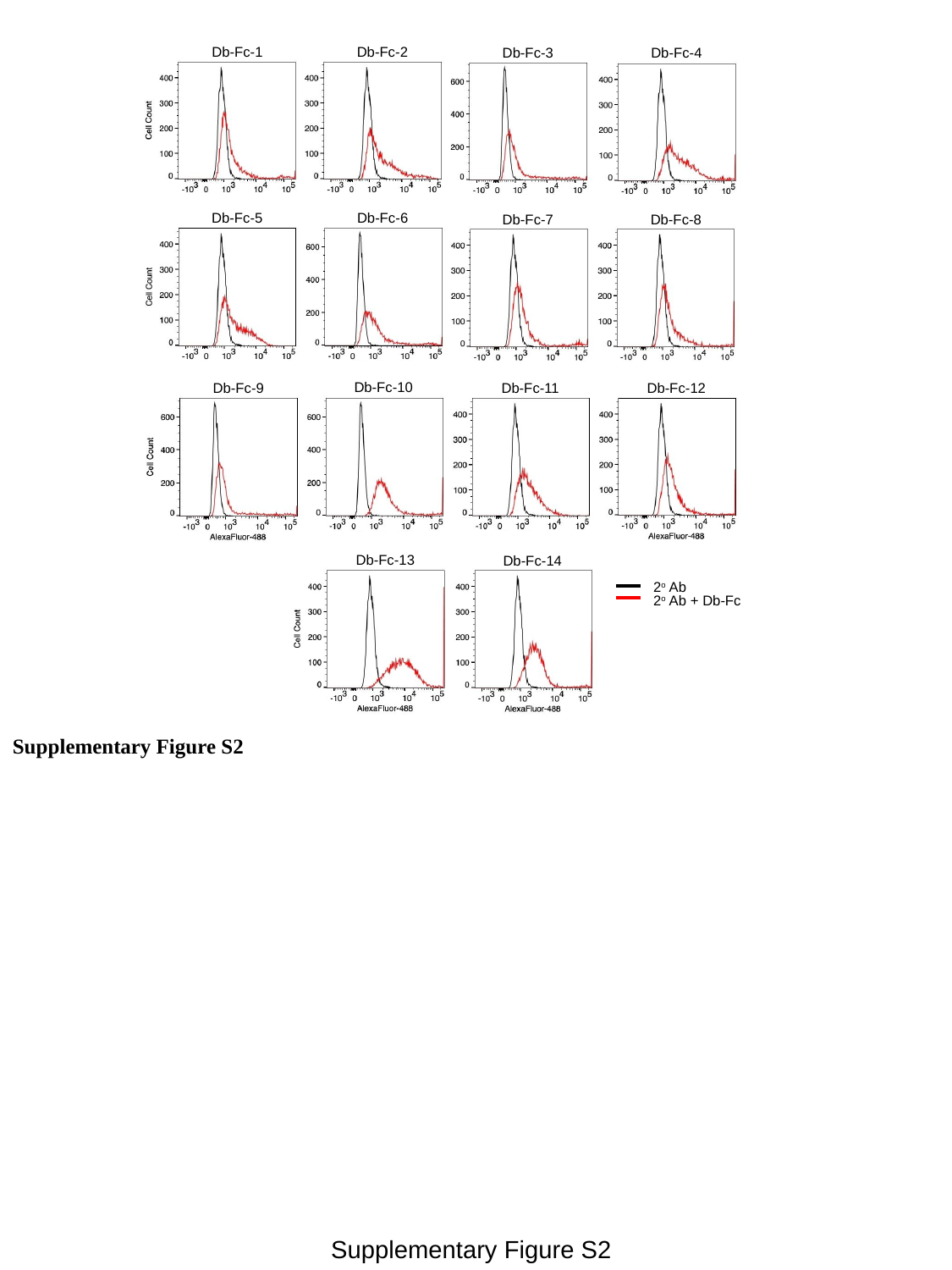

Db-Fc-1
Db-Fc-2
Db-Fc-3
Db-Fc-4
Db-Fc-5
Db-Fc-6
Db-Fc-7
Db-Fc-8
Db-Fc-10
Db-Fc-12
Db-Fc-9
Db-Fc-11
Db-Fc-13
Db-Fc-14
2o Ab
2o Ab + Db-Fc
Supplementary Figure S2
Supplementary Figure S2

## Slide 3
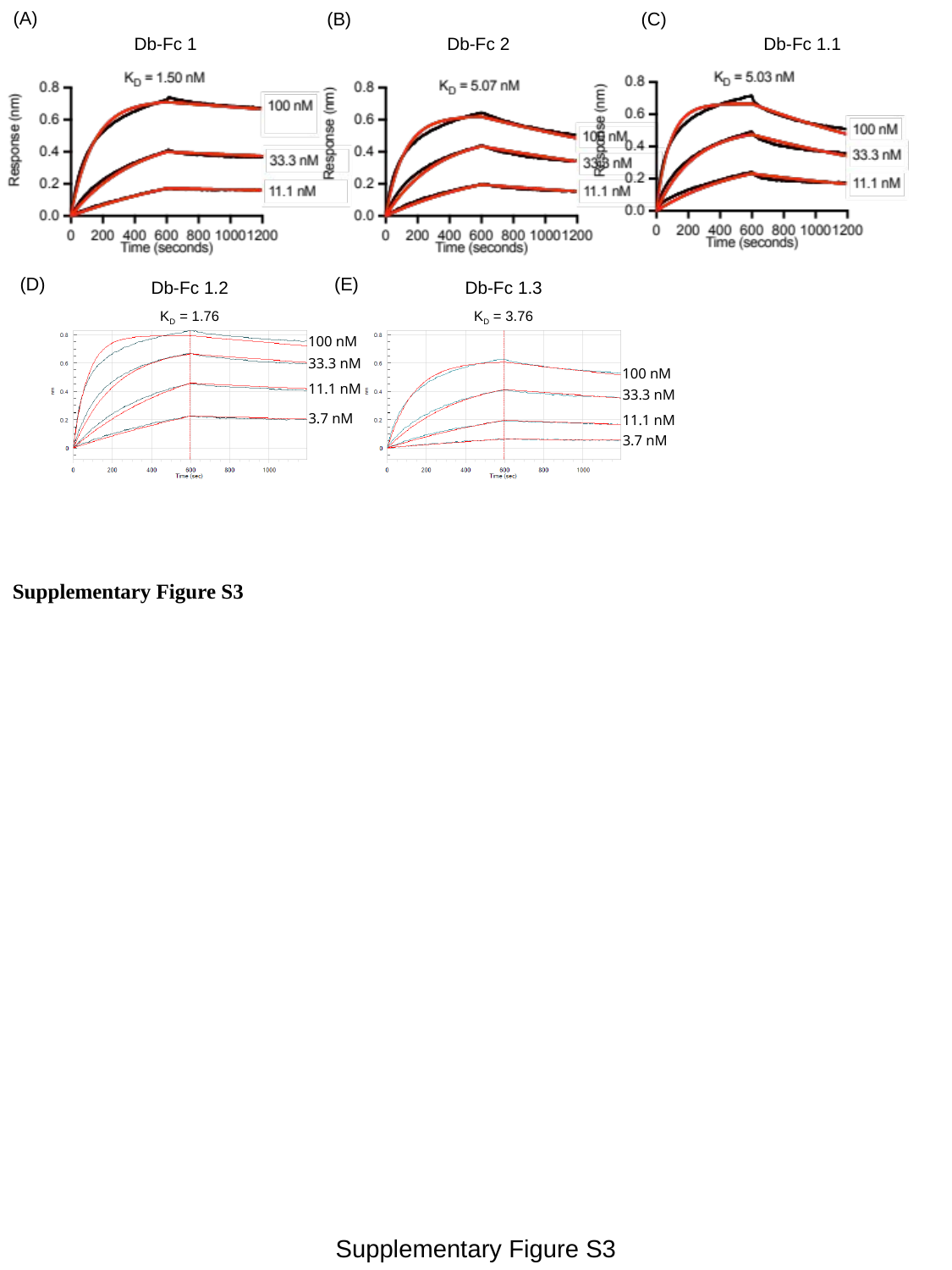

(A)
(B)
(C)
Db-Fc 1.1
Db-Fc 1
Db-Fc 2
(D)
(E)
Db-Fc 1.2
Db-Fc 1.3
KD = 1.76
KD = 3.76
100 nM
33.3 nM
100 nM
11.1 nM
33.3 nM
3.7 nM
11.1 nM
3.7 nM
Supplementary Figure S3
Supplementary Figure S3

## Slide 4
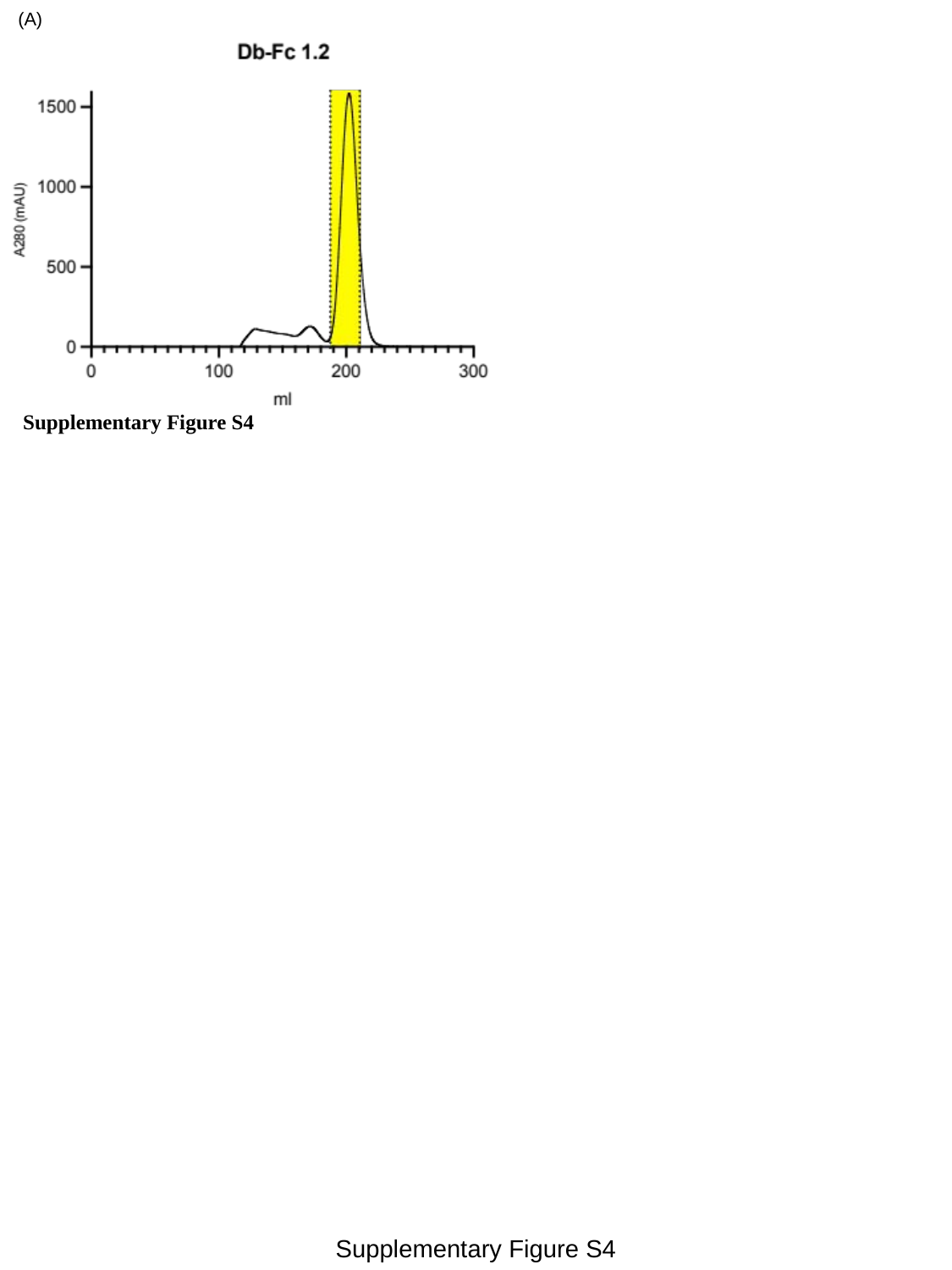

(A)
Supplementary Figure S4
Supplementary Figure S4

## Slide 5
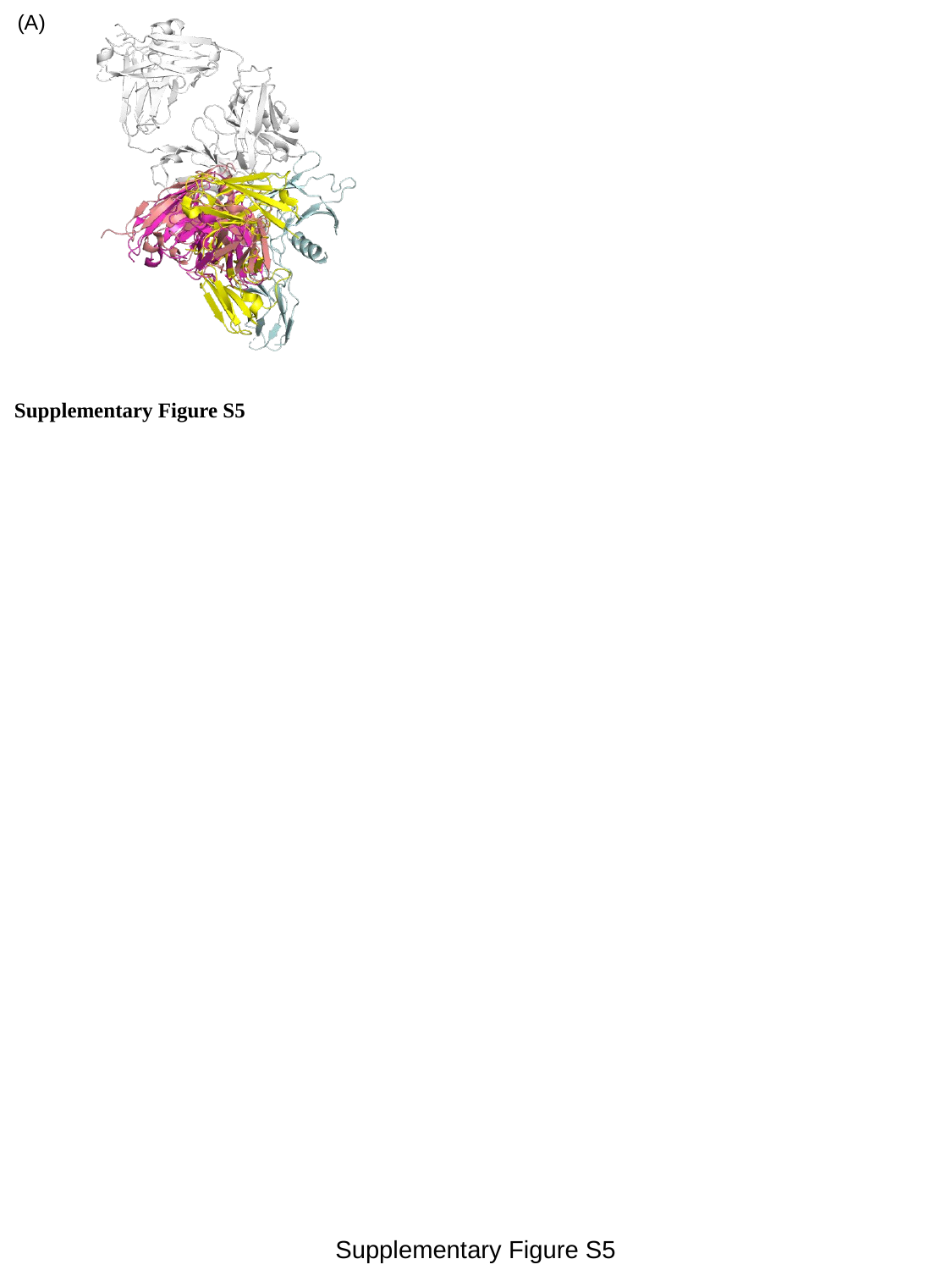

(A)
Supplementary Figure S5
Supplementary Figure S5

## Slide 6
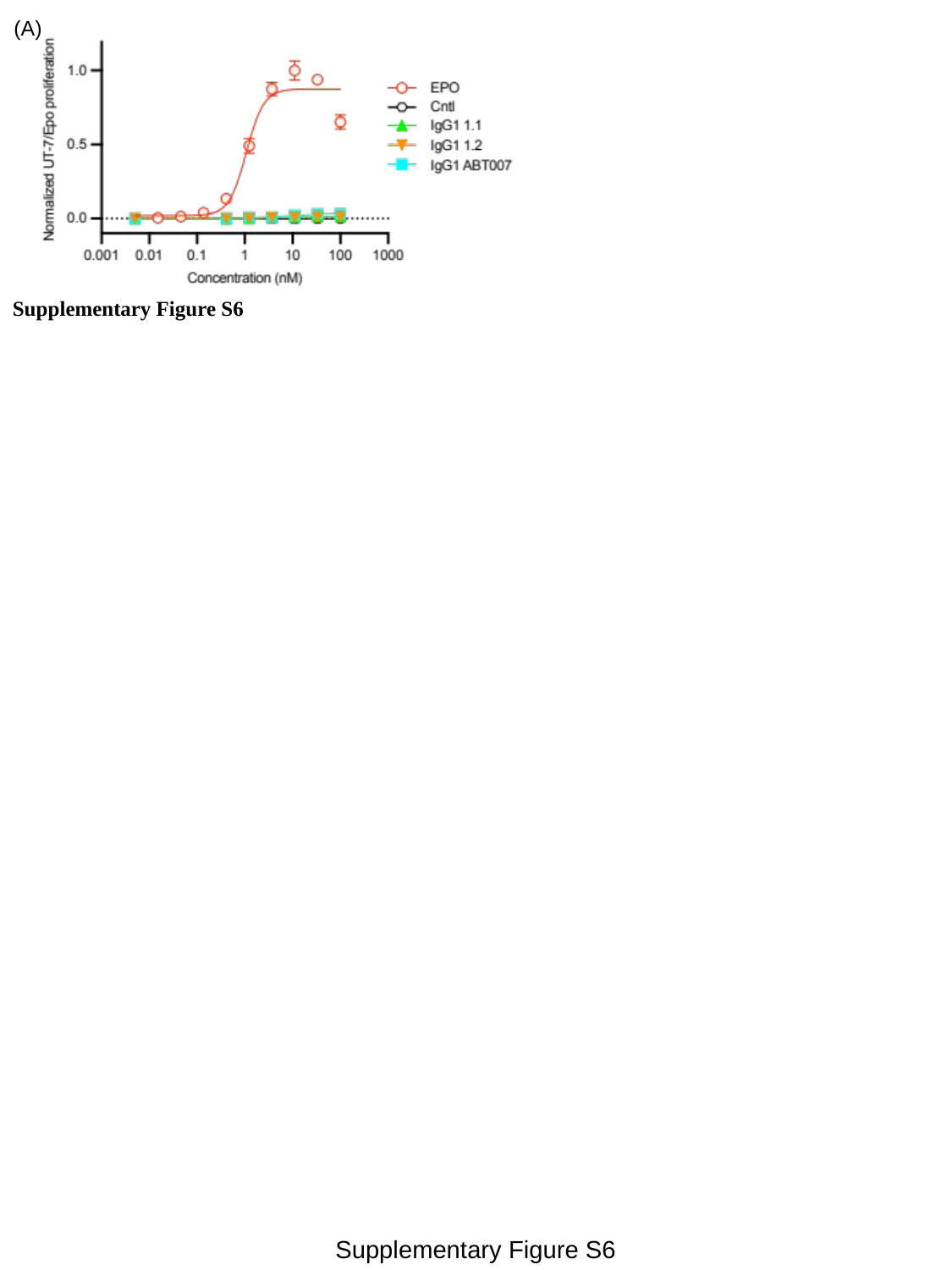

(A)
Supplementary Figure S6
Supplementary Figure S6

## Slide 7
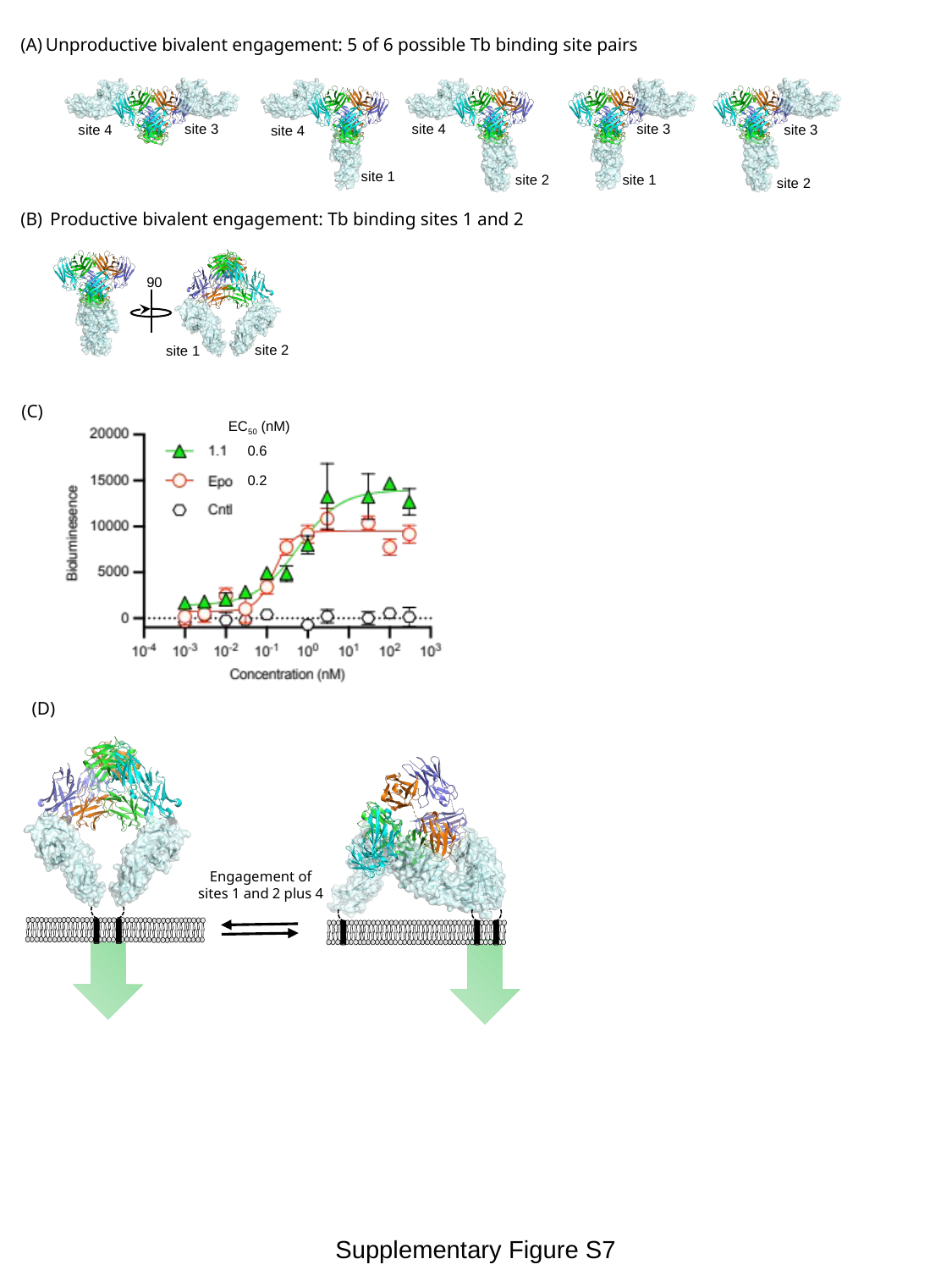

(A)
Unproductive bivalent engagement: 5 of 6 possible Tb binding site pairs
site 4
site 3
site 3
site 4
site 3
site 4
site 1
site 2
site 1
site 2
(B)
Productive bivalent engagement: Tb binding sites 1 and 2
site 2
site 1
(C)
EC50 (nM)
0.6
0.2
(D)
Engagement of
sites 1 and 2 plus 4
Supplementary Figure S7
